# Dermatological Evidence for Coumarins Occurring in Apiaceae: Separating Traditional Use, Botanical Occurrence, and Compound Pharmacology

**DOI:** 10.3390/molecules31213996

**Published:** 2026-11-05

**Authors:** Jiaqi Gou, Ying Zhang, Xuejiao Yang, Yuting Xu, Yixuan Bu, Xiaomi Yang, Jianying Zhang, Aixue Zuo

**Affiliations:** 1 Key Laboratory of Sustainable Utilization of Southern Medicinal Resources, School of Traditional Chinese Medicine, Yunnan University of Chinese Medicine, 1076 Yuhua Road, Chenggong District, Kunming 650500, China; goujiaqi@stu.ynucm.edu.cn (J.G.);; 2 Yunnan Key Laboratory of Integrated Traditional Chinese and Western Medicine for Chronic Disease Prevention and Treatment, Yunnan University of Chinese Medicine, Kunming 650500, China; 3 School of Basic Medical Sciences, Yunnan University of Chinese Medicine, 1076 Yuhua Road, Chenggong District, Kunming 650500, China

**Keywords:** ethnopharmacology, psoralen plus ultraviolet A (PUVA), methoxsalen, osthol, imperatorin, *Cnidium monnieri*, phototoxicity, skin pharmacokinetics, atopic dermatitis, psoriasis

## Abstract

Apiaceae plants have documented uses for pruritic and inflammatory skin conditions. Their coumarins include clinical photosensitizers and experimental candidates, but botanical occurrence alone does not establish equivalence to traditional preparations. To assess dermatological evidence for Apiaceae coumarins and identify where traditional use, tested material, skin exposure, and photosafety support further development, this narrative review drew on PubMed and PubMed Central records, targeted web searches, citation checking, guidelines, and regulatory sources; documented search and update activities took place in July and September 2026. Studies were compared by disease, material, route, comparator, skin exposure, and photosafety. Controlled psoralen–ultraviolet A (UVA) treatment has established clinical use. An authenticated *Cnidium monnieri* fruit extract and its isolated osthol provide a material-specific experimental link. Osthol reduced dermatitis and scratching in mouse models. Imperatorin reduced psoriasiform changes after systemic dosing and rosacea-like inflammation after topical application in mice. Umbelliferone slightly altered pigmentation measures in 12 healthy volunteers and improved oral-dose wound closure in diabetic mice. Comparative topical efficacy at measured skin concentrations remains unresolved. Clinical evidence is concentrated in controlled photochemotherapy; support for other coumarins remains experimental. Further development requires chemically defined materials, appropriate comparators, measured skin exposure, and formulation-specific photosafety. Constituent activity alone does not validate traditional use; linking these results to traditional preparations requires preparation-matched comparisons of extracts and constituents.

## 1. Introduction

Skin and subcutaneous diseases, excluding sexually transmitted infections and skin cancers, affected an estimated 25.7% of the global population in the Global Burden of Disease 2021 analysis [1]. Clinical goals differ across these conditions: relief of itch, restoration of the barrier, stable repigmentation, wound closure, and durable tumor control require different evidence.

Dermatological endpoints are not interchangeable. A reduction in cytokine expression may accompany relief of inflammation without restoring the epidermal barrier; reduced melanin production has a different meaning from repigmentation in vitiligo. Wound closure and durable tumor control require disease-specific models and comparators [2,3,4].

Relapse, incomplete response, and treatment burden remain concerns in inflammatory and pigmentary disease [4,5]. Chronic wounds and advanced melanoma pose different problems, including failure to heal and treatment resistance [2,3]. A new natural-product intervention therefore needs a defined role within the treatment pathway for its intended disease.

Apiaceae coumarins already provide an established dermatological treatment and several compounds for further study. Psoralen plus ultraviolet A (UVA) therapy, known as PUVA, is used for psoriasis, selected cases of vitiligo, and early mycosis fungoides [6,7,8]. Methoxsalen is the principal clinical photosensitizer, and bergapten is used in selected settings [9,10]. Osthol and imperatorin lack clinical evidence but have shown disease-related activity in models of atopic dermatitis, pruritus, psoriasis, and rosacea. Related furanocoumarins can also injure skin after light exposure. The therapeutic evidence for PUVA and the experimental candidates therefore needs to be assessed separately from the photosafety of furanocoumarin-rich products.

Previous reviews cover coumarin pharmacology, furanocoumarin toxicity, Apiaceae antioxidants, skin aging, autoimmune mechanisms, and the therapeutic potential of esculetin [11,12,13,14,15,16,17]. The contribution of this review is a disease-specific assessment of how a reported effect relates to the material actually administered, its botanical context, route, comparator, exposure in skin, and light-dependent risk. We distinguish evidence for a traditional preparation from evidence for a chemically defined constituent and identify the experiments needed to connect them.

## 2. Literature Selection and Evidence Assessment

### 2.1. Design, Information Sources, and Documented Searches

The reporting of this narrative review follows the Scale for the Assessment of Narrative Review Articles (SANRA) criteria [18]. The review was not conducted as a systematic review; a Preferred Reporting Items for Systematic Reviews and Meta-Analyses (PRISMA) flow diagram, pooled estimates, and a formal risk-of-bias assessment are therefore not presented. The evidence base is a narrative literature collection that was assembled in stages, supplemented by targeted searches and by full-text checking of selected studies. PubMed and PubMed Central records were the main bibliographic sources. Other sources were web searches, OpenAlex-assisted discovery, relevant reviews and their reference lists [11,12,13,14,15,16,17], publisher websites, clinical guidelines and consensus statements, pharmacopoeial monographs, and regulatory documents. Search concepts combined Apiaceae or representative genera with coumarins or named compounds, skin diseases, administration routes, exposure, and photosafety.

Two dated searches are documented in the project records. On 27 July 2026, a title-level PubMed search for reviews on the same topic combined coumarin or furocoumarin with skin, dermatology, psoriasis, vitiligo, or dermatitis and returned four records; its full Boolean string, field tags, and filters were not retained. On 5 September 2026, a targeted update used web searches and OpenAlex-assisted discovery, compared this review with ten reviews on the same topic, and checked selected primary studies; it was not an exhaustive repeat search. The complete query parameters for the collection as a whole and the screening decision for each record were not retained, so the numbers of records identified, screened, and excluded are not reported. Supplementary Tables S1–S3 describe traditional-material links, nomenclature, and the evidence map; Table S4 records the studies given detailed appraisal; and Table S5 records the documented searches and evidence-acquisition steps with their limitations, together with a search strategy compiled afterwards to set out the search concepts, whose historical execution could not be confirmed.

### 2.2. Scope Criteria and Literature Screening

The following scope criteria define the evidence considered in this review: (i) the study material was a coumarin reported to occur in Apiaceae, an Apiaceae extract or preparation containing coumarins, or, only as an explicitly labelled comparator, a related non-Apiaceae coumarin or the furanochromone khellin; (ii) the study addressed human treatment or safety, skin pharmacokinetics, an animal model of skin disease, organotypic or ex vivo skin, a defined skin- or sensory-cell system, formulation, or phototoxicity; (iii) the administered material, route, and dermatological endpoint were identifiable from the report; and (iv) the source was a peer-reviewed primary report, a clinical guideline or consensus statement, a pharmacopoeial monograph, or a regulatory document. Botanical extracts were retained as extract-level evidence when the reported material and dermatological experiment were identifiable, even when the active constituent was unresolved. Reviews were used for context and citation checking, not as primary evidence of efficacy.

Studies were not used as evidence when they were general antioxidant or enzyme screens without a skin-relevant model, docking-only or other purely in silico studies, or experiments without a dermatological endpoint or connection, or when the test material could not be identified. Where the same dataset was reported more than once, the most complete report was used. A mixture effect was not assigned to an individual coumarin without a corresponding constituent experiment. These criteria describe the scope of the evidence considered; the number of reports excluded under each criterion was not retained. Literature screening was performed by two authors (J.G. and Y.Z.), assisted by the generative artificial intelligence (AI) tool described in Section 2.5.

### 2.3. Study-Level Appraisal and Risk of Selection Bias

Included primary studies were assessed for the material, route, model, comparator, and endpoints summarized in the study tables, from the full text where it was accessible. A more detailed study-level check (Table S4) was carried out on a set of primary reports that was assembled in stages. The set first comprised ten reports; eight were assessed from their main texts, and two (Hu et al. [19] and Tsai et al. [20]) were at first available only at abstract level. The full texts of these two reports were then obtained and assessed. The full text of Khalil et al. [21] was subsequently obtained, assessed, and added to the set, giving eleven reports: Matsuda et al. [22], Yang et al. [23], Hu et al. [19], Kordulewska and Król-Grzymała [24], He et al. [25], and Naeem et al. [26] (osthol); Tsai et al. [20] and Wang and Zhu [27] (imperatorin); the two studies by Kim et al. [28,29] (umbelliferone); and Khalil et al. [21] (esculetin). The number eleven therefore reflects this stepwise process; it was not set in advance, and the set was not selected from a complete pool of candidate studies by a predefined rule. Other studies, including those of Lim et al. [30] on umbelliferone and Chen et al. [31] on esculetin, were described with the same descriptors, but without the additional checks listed below. PUVA studies were not included in this set; the clinical role of PUVA is based on guidelines, controlled trials, and long-term cohort data (Section 5.1).

The heterogeneous diseases, outcomes, routes, and models precluded a pooled effect estimate. The review does not claim exhaustive retrieval or a formal risk-of-bias assessment. For the eleven studies detailed in Table S4, primary full texts were inspected for dose, route, comparator, sample size, allocation or blinding, and the relationship between measured endpoints and proposed use. Details absent from an inspected report were distinguished from information in inaccessible Supplementary Files or other references available only at abstract level. Selective retrieval and incomplete reporting may affect the resulting priorities.

Null, negative, and inconclusive findings identified in the collection are reported together with positive ones. Examples include the lack of effect of osthol on chloroquine-evoked scratching and the non-significant effect of one esculetin carrier formulation on tumor necrosis factor-alpha (TNF-alpha) (Section 5.2 and Section 6), the absence of a significant difference between bath and systemic PUVA and the non-significant intention-to-treat result in palmoplantar pustulosis (Section 5.1), negative phototoxicity results for isopimpinellin and bergamottin (Section 4.4), and a published correction to a decursinol angelate study (Section 5.6). Reporting these findings does not exclude selection bias. The collection was assembled narratively; its composition depended in part on which full texts could be obtained, and relevant studies may not have been retrieved. Each conclusion is therefore limited to the material, route, and model that were tested, and the resulting priorities are conditional (Section 8.3).

### 2.4. Levels of Evidence and Candidate Assessment

Four levels of evidence were kept separate. Documented traditional use refers to a named species, plant part, preparation, route, and indication recorded in a pharmacopoeial monograph, a historical or ethnobotanical source, or a review citing such sources. Botanical occurrence refers to detection or isolation of a constituent in an identified taxon; it does not establish the concentration of that constituent in a traditional preparation or its activity. Extract-level activity refers to a dermatological effect of an extract whose botanical identity and preparation are reported, and is interpreted according to the extract-characterization principles of the Consensus statement on the Phytochemical Characterisation of Medicinal Plant extracts (ConPhyMP) guidelines [32]. Isolated-compound pharmacology refers to an effect of a chemically defined compound, whether it was isolated from a plant, purchased, or synthesized. A finding at one level was not taken as evidence at another: activity of a compound was not taken to validate the traditional use of a plant that contains it, and the occurrence of a compound in a plant was not taken to show that preparations of that plant are active. Supplementary Table S1 assigns these levels to the principal materials.

Candidate selection follows three questions: does the administered material improve a relevant disease endpoint; can the route and exposure plausibly support the intended use; and what evidence would change that judgment? Complementary models increase biological plausibility but do not constitute independent replication of the same experiment, particularly when reports share investigators. Route, active comparators, and source-verification limits qualify each priority; no numerical or comparative efficacy ranking was calculated. Chemical identity and purity are assessed separately from botanical provenance. The evidence map displays evidence coverage and identified gaps rather than study quality or clinical readiness.

### 2.5. Use of Generative Artificial Intelligence

OpenAI Codex (OpenAI), using GPT models including GPT-5.6 Sol, GPT-5.6 Terra, GPT-6 Astra, GPT-6.1 Sol, and GPT-6.1 Terra, was used at different stages of manuscript preparation and proofreading to assist with literature searching and screening (including web searches, OpenAlex-assisted discovery, and checking of sources), language revision, document formatting, organization of literature-derived material, and preparation of explanatory schematics, a qualitative literature-evidence map, and their labels. An OpenAI image-generation tool was used to prepare preliminary figure drafts; its specific model version could not be determined from the available records. The figures summarize the reviewed literature and a proposed development workflow. The authors reviewed the AI-assisted content; the manuscript text was revised, and the figures were redrawn and finalized in Adobe Illustrator. No primary experimental images, measurements, or observations were generated using these tools. The authors take full responsibility for the final manuscript. Figure-specific uses are disclosed in the corresponding legends. Literature identified with the tool was screened by two authors (J.G. and Y.Z.).

## 3. Clinical Objectives and Experimental Comparators

Table 1 defines the clinical objective against which each experimental finding should be interpreted. The following context is limited to features that change the choice of model, endpoint, or comparator.

### 3.1. Atopic Dermatitis and Chronic Pruritus

Atopic dermatitis affects children, adolescents, and adults [33]. Its pathophysiology combines epidermal barrier dysfunction and immune activation [34]. Interleukin (IL)-4, IL-13, and IL-22 can alter epidermal barrier programs, so suppression of one inflammatory signal need not restore barrier function [34]. Topical anti-inflammatory treatment is the relevant reference for a local candidate; systemic biologics and Janus kinase (JAK) inhibitors address disease requiring escalation [5,35]. Inflammation, barrier function, and scratching should be evaluated separately. Chronic prurigo is a distinct indication, with its own phototherapy and targeted-treatment recommendations [36].

### 3.2. Psoriasis and Palmoplantar Inflammatory Disorders

The IL-23/IL-17 axis connects immune activation to keratinocyte responses in psoriasis [37,38]. Imiquimod and IL-23 models test selected components of this biology; they do not establish comparative clinical efficacy. A topical candidate should be assessed against an appropriate local treatment, while PUVA is relevant to selected patients requiring phototherapy [7]. Palmoplantar eczema and pustulosis require separate evaluation because their presentation and phototherapy evidence differ from plaque psoriasis [39,40,41].

### 3.3. Rosacea

Rosacea includes inflammatory, vascular, sensory, ocular, and phymatous manifestations. Macrophage-associated Toll-like receptor 2 (TLR2) and cathelicidin pathways address only part of that spectrum [42]. The model induced by the human cathelicidin peptide LL-37 used for imperatorin is an acute inflammatory challenge and should be interpreted against inflammatory-lesion treatment rather than persistent telangiectasia or ocular disease. Clinical comparisons must specify the target phenotype [43].

### 3.4. Vitiligo and Other Pigment Disorders

Vitiligo treatment aims to arrest melanocyte loss and restore stable pigment. Topical anti-inflammatory agents, ruxolitinib, and narrowband ultraviolet B (UVB) provide relevant clinical benchmarks [4,44,45]. A compound that reduces melanin in B16F10 cells addresses a different question. Hyperpigmentation studies require a defined diagnosis and pigment-reduction endpoint; neither melanogenesis inhibition nor cosmetic brightening demonstrates efficacy in autoimmune vitiligo.

### 3.5. Impaired Wound Repair

Chronic diabetic ulcers reflect impaired repair together with pressure, neuropathy, perfusion deficits, infection, and metabolic disease. Standard care addresses these contributors through measures such as offloading, debridement, perfusion management, and infection treatment [3]. A scratch assay measures a restricted repair process. A diabetic mouse excision model adds an in vivo closure endpoint, but does not reproduce a chronic human foot ulcer or establish benefit when added to standard care.

### 3.6. Melanoma

Melanoma treatment depends on stage, resectability, and molecular features. Surgery, anti-programmed cell death protein 1 (PD-1) therapy, and B-Raf serine/threonine kinase (BRAF)–mitogen-activated protein kinase kinase (MEK) inhibition in eligible tumors define different clinical settings [2]. Cell viability or tumor-volume changes in experimental systems are early pharmacological findings; they do not show activity after clinical resistance, durable disease control, or tolerable exposure in patients.

### 3.7. Early Mycosis Fungoides and Other Selective Uses of PUVA

PUVA has a selected skin-directed role in early mycosis fungoides, particularly for plaques [8,46]. Response duration and cumulative exposure therefore matter alongside initial clearance. Bath-PUVA series and long-term safety observations are discussed in Section 5.1 [47,48].

**Table 1:** Clinical objectives and evidence interpretation for Apiaceae-associated treatments and compounds.

Disease Setting	Relevant Disease Process	Current Treatment Reference	Unresolved Need Relevant to a New Candidate	Interpretation of Candidate Evidence
Atopic dermatitis and chronic pruritus	Epidermal barrier dysfunction and type 2/IL-22 inflammation [34]	Skin care and topical therapy; biologics, JAK inhibitors, and selected phototherapy or systemic agents [5,35]	Durable control of inflammation and pruritus with acceptable local or systemic safety	Topical osthol reduced oxazolone dermatitis and scratching [19]; comparative efficacy of defined topical products remains unestablished.
Psoriasis	IL-23/IL-17-driven keratinocyte inflammation [38]	Topical treatment, narrowband UVB, conventional systemic drugs, and targeted biologics [37]	Effective local treatment or a defined role when systemic treatment is unsuitable	Methoxsalen-PUVA is clinically established; imperatorin evidence is limited to disease models, with topical exposure and UVA safety still unknown
Palmoplantar inflammatory disorders	Site-specific hyperkeratotic or pustular disease	Selected phototherapy; evidence and schedules remain limited [39,41]	Better evidence for route, schedule and durable response	PUVA is a selective option; no individual Apiaceae compound can yet replace it, and disease-specific comparative trials are needed
Rosacea	Phenotype-dependent innate immune, vascular and sensory abnormalities	Anti-inflammatory, antimicrobial or vascular treatment chosen by phenotype [43]	Durable control without irritation or rebound and with a clear target phenotype	Imperatorin has a short vehicle-controlled topical result during LL-37 induction; established-disease treatment and clinical benefit remain untested.
Vitiligo	Autoimmune melanocyte loss	Topical corticosteroids, calcineurin or JAK inhibition, and narrowband UVB [4,44]	Stable and durable repigmentation with manageable treatment burden	PUVA has selected historical use. The non-PUVA pigment-reduction studies do not test melanocyte preservation or repigmentation in vitiligo.
Hyperpigmentation	Excess or uneven pigment production, depending on diagnosis	Diagnosis-specific topical and procedural care	Clinically meaningful, durable pigment change without irritation	Umbelliferone has a four-week vehicle-controlled study in 12 healthy adults; disease-specific benefit and persistence remain unresolved.
Diabetic wound	Pressure, perfusion, infection, and impaired tissue repair	Multidisciplinary standard care [3]	Adjunctive benefit in wounds that fail to heal	Oral umbelliferone improved closure in diabetic mouse excisional wounds. Benefit in chronic human ulcers or added to standard care remains unestablished.
Melanoma	Stage- and mutation-defined malignant disease	Surgery, anti-PD-1-based treatment, and BRAF-MEK therapy when indicated [2]	Activity after resistance or in a defined molecular setting	Decursin-class compounds and umbelliprenin remain discovery-stage findings without a clinically relevant comparison
Early mycosis fungoides	Cutaneous T-cell lymphoma	Skin-directed treatment including selected PUVA [8,46]	Durable response with controlled cumulative exposure	Methoxsalen-PUVA has clinical support; lower-burden skin-directed protocols and long-term safety remain priorities

BRAF, B-Raf serine/threonine kinase; IL, interleukin; JAK, Janus kinase; LL-37, human cathelicidin peptide; MEK, mitogen-activated protein kinase kinase; PD-1, programmed cell death protein 1; PUVA, psoralen plus ultraviolet A; UVA, ultraviolet A; UVB, ultraviolet B.

Defined psoralen–UVA protocols occupy selected clinical roles. The other compounds were studied mainly in cells or animals, with few measurements of viable-skin exposure or formulation-specific light response. Several purified materials had a documented commercial identity but no link to an authenticated botanical batch; that limits extrapolation to a plant preparation, rather than invalidating a chemically defined pharmacological result.

## 4. Ethnopharmacological Context, Plant Sources, and Coumarin Chemistry

Apiaceae includes medicinal plants, food crops, and aromatic species in genera such as *Angelica*, *Cnidium*, *Peucedanum*, *Heracleum*, *Pastinaca*, *Ammi*, and *Ferula*. Reported constituents include linear furanocoumarins, oxygenated simple coumarins, dihydropyranocoumarins, and sesquiterpene coumarins [15,49]. Psoralens can serve as medicinal photosensitizers; simple coumarins such as osthol are being studied experimentally, and some furanocoumarins cause skin injury after light exposure. Activity of one constituent does not mean that every compound in the same plant or chemical group is effective.

### 4.1. Traditional Skin Uses and the Extract–Constituent Link

*Cnidium monnieri* (L.) Cusson fruit is a useful test of the relationship between traditional use and isolated-compound pharmacology. Matsuda et al. described its use in Chinese medicine for pruritic, suppurative dermatitis and tested a 70% ethanol extract alongside osthol isolated from the same fruit material [22]. A voucher specimen of the fruit material was deposited at Kinki University (now Kindai University), Osaka, Japan, under specimen number CM-99001, and the isolate was characterized spectroscopically [22]. CM-99001 is the specimen number assigned in that study; it does not denote the Index Herbariorum acronym CM, which refers to the Carnegie Museum of Natural History. Oral extract and osthol reduced contact-hypersensitivity ear swelling, with pharmacological reference treatments included. The extract also reduced scratching; that endpoint should not be assigned to isolated osthol from this experiment. The paired design links the botanical material to a constituent for selected outcomes, without establishing topical clinical efficacy.

The preparation tested in that study differs from the preparation recorded for traditional use. In the Chinese Pharmacopoeia, Cnidii Fructus is the dried ripe fruit of *C. monnieri*; its indications include eczema and pruritus, and for external use an appropriate amount is applied, mostly as a decoction used for fumigation and washing or as a powder mixed into a paste [50] (documented traditional use). The Matsuda study instead administered a 70% ethanol extract orally, which differs from the traditional external preparation in extraction solvent and route of administration (extract-level activity) [22]. The osthol content of an aqueous decoction or wash, and the amount reaching the skin from such a preparation, were not measured in the studies reviewed here. Traditional preparations were dosed by the weight of crude drug rather than by constituent content. The 2020 pharmacopoeial monograph specifies identification tests and a minimum osthol content of 1.0% in the dried fruit [50]; this standard applies to the starting material and does not define the amount of osthol in a particular decoction, wash, or paste. A comparison of the traditional preparation applied to skin with the isolated compound at a matched osthol dose, with the constituent profile of each preparation reported, would allow this link to be examined directly.

*Ammi majus* L. is an example of a traditional preparation that was later developed into a defined clinical treatment. In Egyptian folk medicine, powdered *A. majus* fruit was taken orally before sun exposure to treat leukoderma (documented traditional use). Methoxsalen (8-methoxypsoralen; 8-MOP) and related furanocoumarins were isolated from the fruit in the 1940s, after which treatment moved from crude plant material to a defined compound given at a specified dose with controlled light exposure [51]. The clinical evidence summarized in Section 5.1 applies to that defined drug–UVA regimen (isolated-compound pharmacology), not to the original crude preparation, in which psoralen content and light exposure were not standardized.

For *Angelica dahurica* (Hoffm.) Benth. & Hook.f. ex Franch. & Sav., the ethnomedical connection is less direct. A review of traditional uses describes external application of the root for boils, carbuncles, sores, and painful swellings, citing pharmacopoeial sources [52]. The pharmacopoeial monograph for Angelicae Dahuricae Radix lists sores and painful swellings among its indications and specifies a minimum imperatorin content of 0.080% in the dried root, but it gives a dose only in grams of crude drug and does not describe a topical preparation [50]. These sources provide context for inflammatory skin research (documented traditional use); they do not validate a purified imperatorin formulation or equate these historical indications with modern psoriasis or rosacea. The preparation, route, composition, and clinical phenotype need to be matched before making that extrapolation.

Traditional use is consequently evaluated at the species–organ–preparation–indication level (Section 2.4). Some compounds in this review have disease-related pharmacology but no documented traditional use for the modeled condition. They remain compound-discovery findings. Supplementary Table S1 summarizes the selected material links and the limits of inference.

Botanical nomenclature and source-specific names are recorded in Supplementary Table S2, with database records consulted on 5 September 2026. *Ferula szowitziana* DC. is the spelling listed in the World Flora Online (WFO) genus record; the cited efficacy paper uses *Ferula szowitsiana*. This nomenclatural difference does not change the provenance reported by that study. The accepted spelling, *F. szowitziana*, is used throughout the text and Supplementary Tables; the original spelling is retained only in the title of the cited melanoma study.

### 4.2. Structures Relevant to Therapeutic Action and Photosafety

In linear furanocoumarins, fusion of a furan ring to the coumarin nucleus forms the psoralen structure. After absorbing UVA, psoralens can form pyrimidine photoadducts and deoxyribonucleic acid (DNA) interstrand crosslinks [11]. This reaction can suppress hyperproliferative skin cells, but it can also cause erythema and increase genotoxic and carcinogenic risk. Methoxy position, side-chain substitution, lipophilicity, vehicle, route and UVA fluence all affect the response.

**Figure 1:**
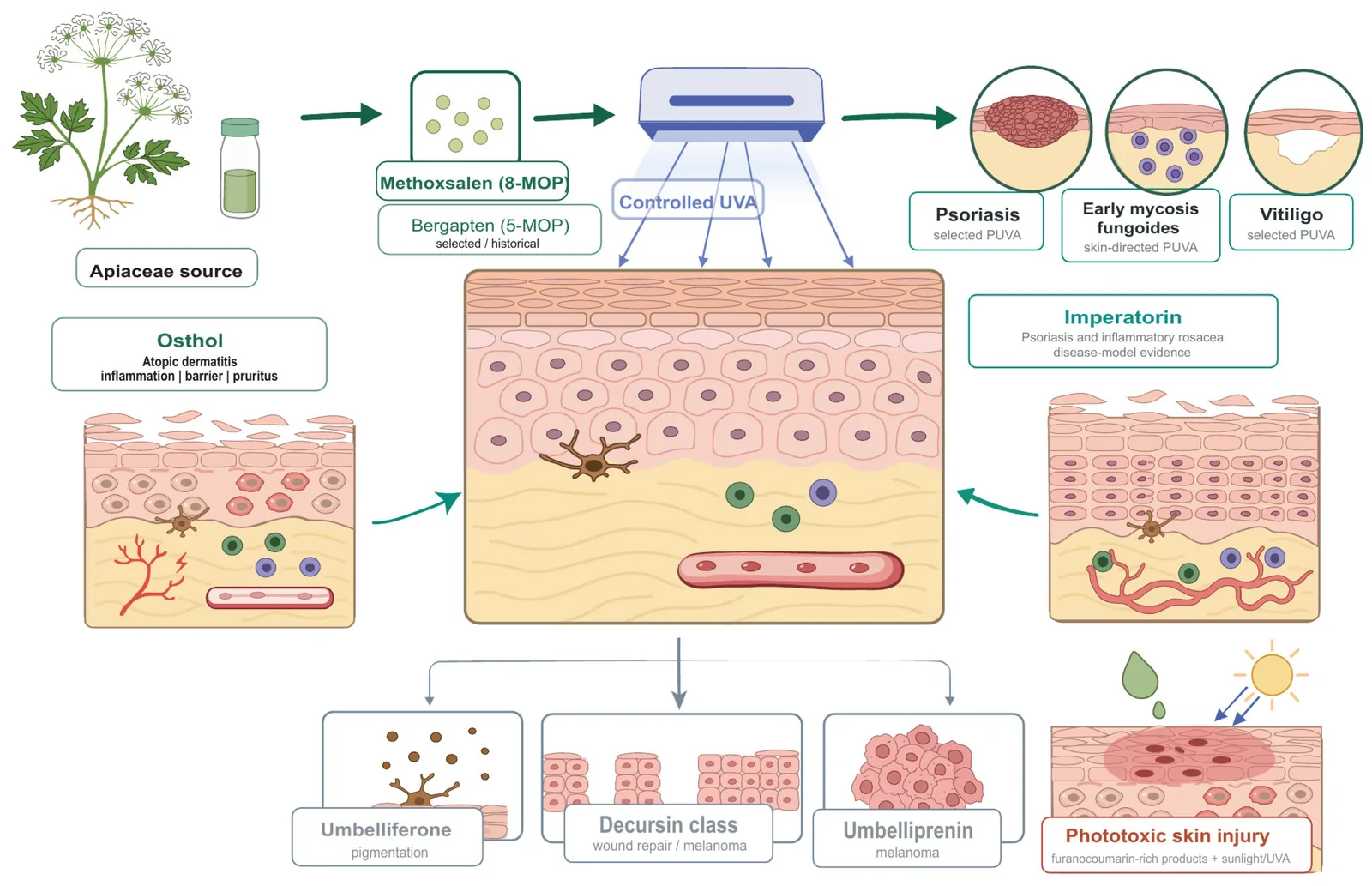
Therapeutic uses and phototoxic exposures of Apiaceae coumarins in dermatology. Methoxsalen (8-MOP) with controlled UVA has established or selected clinical roles in psoriasis, early mycosis fungoides, and vitiligo; bergapten (5-MOP) has more limited, selected or historical use. Osthol and imperatorin are priority preclinical candidates. Evidence for umbelliferone, decursin-class compounds, and umbelliprenin remains preliminary. Furanocoumarin-rich products can cause phototoxic skin injury after sunlight or UVA exposure. OpenAI GPT (see Section 2.5) assisted with preparation of this explanatory schematic and its labels. The figure summarizes published therapeutic and phototoxicity evidence. The authors reviewed the AI-assisted content; the illustration was redrawn and finalized in Adobe Illustrator. It contains no primary experimental images or measurements. 5-MOP, 5-methoxypsoralen; 8-MOP, 8-methoxypsoralen; PUVA, psoralen plus ultraviolet A; UVA, ultraviolet A.

Osthol is a prenylated simple coumarin. Umbelliferone, scopoletin, isoscopoletin, and esculetin are oxygenated simple coumarins. Decursin and decursinol angelate are dihydropyranocoumarin esters, and umbelliprenin is a sesquiterpene coumarin. These compounds lack the fused furan ring of the psoralen nucleus; this structural distinction alone does not establish photosafety. Figure 1 summarizes the therapeutic uses, experimental candidates, and phototoxic exposures considered in this review.

### 4.3. Apiaceae Occurrence and Materials Used in Efficacy Studies

Apiaceae species differ in their characteristic coumarins. Roots of *Angelica dahurica* contain imperatorin and isoimperatorin [53], together with oxypeucedanin, phellopterin and related furanocoumarins summarized in a phytochemical review [52], whereas roots of *Angelica gigas* Nakai contain decursin and decursinol angelate [54]. Osthol has been isolated from *Cnidium monnieri* fruit, and both the fruit extract and osthol have shown anti-allergic activity [22]. In the melanoma study, umbelliprenin was isolated directly from *Ferula szowitziana* [55].

Commercial osthol was used in the sensory-itch, oxazolone, and 2,4-dinitrochlorobenzene (DNCB) studies [19,23,25], whereas Matsuda et al. isolated it from authenticated *Cnidium monnieri* fruit [22]. Tsai et al. used imperatorin isolated from *Angelica hirsutiflora*, with purity >97%; Wang and Zhu used commercial imperatorin of >98% purity [20,27]. These material histories support different source attributions. Validation of a botanical preparation also requires a link to its plant part, extraction, composition, and batch.

Several simple-coumarin studies support compound-level rather than Apiaceae-preparation conclusions. Umbelliferone was commercially sourced in the wound and pigmentation studies [28,29], as was esculetin in the lipid-carrier study [21]. Other experiments addressed purified umbelliferone, esculetin, or isoscopoletin [30,31,56]. The scopoletin study used *Lycium barbarum* L. (Solanaceae) extract and isolate in different experimental arms [57]. Auraptene is included only as a non-Apiaceae topical comparator [58]. Khellin from *Visnaga daucoides* Gaertn. (syn. *Ammi visnaga* (L.) Lam.) is a chemically distinct historical UVA comparator [59]. Table 2 summarizes the plant sources, study materials, and dermatological evidence.

### 4.4. Compound-Specific Photoreactivity

Phototoxic potency varies among linear furanocoumarins and depends on the UVA conditions used for testing. Dark cytotoxicity and cytokine assays cannot define the safety margin of a photoreactive compound. Synthetic isopimpinellin was inactive in a chick-skin assay when analytical testing confirmed that highly active psoralen contaminants were present at most in trace quantities [64]. Bergamottin was also negative in a comparison based on Organisation for Economic Co-operation and Development (OECD) Test Guideline 432, which gave positive results for psoralen, 5-MOP, and 8-MOP [65]. A single assay cannot establish clinical safety, and each furanocoumarin requires separate evaluation.

The formulation and efficacy reports considered here do not establish osthol or decursin-class concentrations in viable human epidermis or dermis. Decursin and decursinol angelate also undergo substantial conversion after systemic administration [66,67,68]. Thus, nominal dose and lipophilicity alone do not identify the chemical species or concentration responsible for a local effect.

## 5. Therapeutic Evidence: Established Photochemotherapy and Experimental Candidates

**Figure 2:**
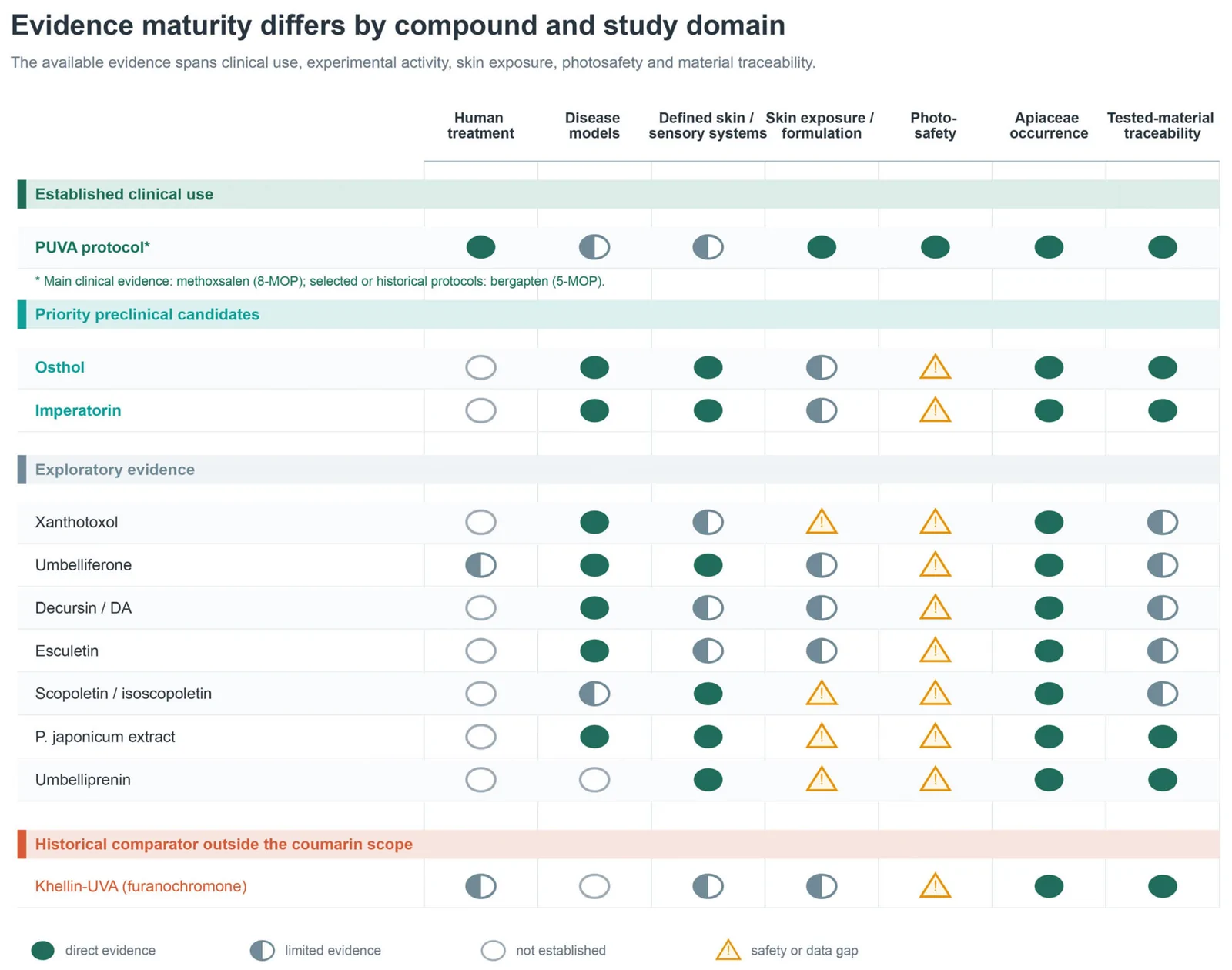
Evidence coverage across Apiaceae-associated treatments and compounds. Columns separate human observations, disease models, defined skin or sensory systems, skin exposure or formulation, photosafety, botanical occurrence, and material traceability. The original symbols distinguish direct, limited, unestablished, or gap-marked evidence within this narrative collection; they are not risk-of-bias grades, effect-size comparisons, or a ranking of clinical readiness. Human umbelliferone evidence concerns healthy-volunteer pigmentation measurements. Scopoletin animal evidence concerns *Lycium* extract. The combined exposure/formulation column includes delivery studies without measured viable-skin concentrations. Material traceability is separate from batch-level botanical authentication. Supplementary Table S3 and its CSV give domain-specific references and qualifications; Table S4 provides selected study-level checks. OpenAI GPT (see Section 2.5) assisted with preparation of this qualitative literature-evidence map and its labels. Its symbols summarize the evidence coverage and gaps described in the reviewed literature and do not represent primary experimental measurements. The authors reviewed the AI-assisted content; the figure was redrawn and finalized in Adobe Illustrator.

Defined psoralen-UVA treatment accounts for the established clinical evidence (Table 3). Non-PUVA findings range from cell assays to animal disease models and a small pigmentation study in healthy volunteers (Table 4). Figure 2 provides a qualitative overview; study-level details in Table S4 qualify the osthol and imperatorin priorities and the interpretation of umbelliferone and esculetin.

### 5.1. Established Psoralen Photochemotherapy

Methoxsalen-PUVA is the established Apiaceae-coumarin treatment in psoriasis. A foundational oral PUVA study described a workable regimen [69], and later work showed that exposure varies with psoralen identity, formulation, food intake, and patient factors [10,70]. Clinical guidance treats methoxsalen as a photosensitizing drug administered with controlled UVA [9]. Bergapten has also been used, but the clinical literature is older and less extensive [9,10]. Clinical evidence for these drugs does not extend to psoralen-containing plant preparations.

A PUVA treatment protocol specifies the psoralen, route, interval before irradiation, initial UVA dose, increments, protection, and follow-up. Ultraviolet (UV) treatment affects keratinocytes, lymphocytes, antigen presentation, and inflammatory programs in addition to producing psoralen photoadducts [71]. Oral, bath, and cream delivery produce different exposure profiles. In an eight-person crossover study, bath and cream administration gave higher, more predictable skin concentrations of 8-MOP and substantially lower plasma exposure than oral dosing [72]. In a randomized, multicenter study, bath and systemic PUVA both reduced the Psoriasis Area and Severity Index (PASI) in 74 patients with moderate-to-severe plaque psoriasis. The routes did not differ significantly after six weeks [73]. Topical protocols still require control of psoralen concentration and distribution, application-to-irradiation timing, and UVA dose [74].

Narrowband UVB is now the usual first-line phototherapy for many patients because it avoids psoralen and is easier to deliver. PUVA remains an option when disease thickness, prior response, availability, or failure of another phototherapy justifies the additional burden [7,9,75]. Long-term exposure is the central safety constraint. In the 30-year PUVA Follow-Up Study, 350–450 treatments were associated with an incidence rate ratio of 6.01 for at least one annual squamous cell carcinoma [76]. The reference group received fewer than 50 treatments. When all tumors were counted, the incidence rate ratio was 20.92. A separate systematic review found greater cumulative carcinogenic concern for PUVA than for narrowband UVB [77]. A 2026 prospective cohort included 20 patients with plaque psoriasis and a recent internal malignancy [78]. Mean PASI fell by about 69%, with weaker responses on the lower limbs. The study had no comparator, so the result applies only to this selected non-immunosuppressive setting.

Studies of palmoplantar disease are small and have yielded inconsistent results. Oral and bath PUVA have been compared in chronic palmoplantar eczema [41]. A 2026 study of palmoplantar pustulosis compared bath PUVA, tap-water-UVA, and saltwater-UVA; the intention-to-treat result was not significant, although the per-protocol analysis was [40]. Current guidance allows consideration of PUVA but does not establish an optimal schedule [39]. Because palmoplantar disorders differ clinically from plaque psoriasis, their results do not strengthen the evidence for plaque psoriasis.

PUVA has historical activity in vitiligo, but narrowband UVB now provides the more relevant phototherapy benchmark. A randomized, double-blind comparison found better repigmentation quality and color match with narrowband UVB than with PUVA [45]. Current guidance similarly favors narrowband UVB [4,6]. Bergapten has been evaluated only in selected PUVA protocols. These protocol-specific findings cannot be extrapolated to crude botanical preparations.

Khellin-UVA provides a historical non-coumarin comparison. An early 28-patient study reported repigmentation in some participants together with liver-enzyme elevations [59]; a separate report described marked transaminase increases after khellin–UVA treatment [63]. A later open study of liposomal khellin with UVA/UVB reported site-dependent responses but lacked blinded randomization [62]. Khellin is a furanochromone, and these small studies do not establish it as a clinical alternative or support coumarins as a class.

Early mycosis fungoides has a guideline-supported clinical role for PUVA [8,46]. A retrospective series of 62 patients reported substantial responses to bath-PUVA [48]. In a separate cohort of 104 PUVA-treated patients, at least 250 sessions were associated with higher skin-cancer risk (hazard ratio 4.44) [47]. Disease-related baseline risk and treatment history complicate that association, but the finding supports cumulative-exposure records and surveillance.

### 5.2. Osthol and Related Candidates for Dermatitis and Pruritus

The osthol literature contains complementary experiments with different routes and endpoints. In oxazolone-induced dermatitis, Hu et al. applied osthol topically at reported doses of 15 or 45 mg/kg twice daily for seven days, beginning with the first challenge; lesion scores and scratching decreased [19]. Each group contained six mice, but the carrier, application volume or area, and whether the stated dose was per application or per day were not specified. No standard active drug was included in that osthol experiment. In the DNCB study, osthol was given at 5, 25, or 50 mg/kg by gavage, as identified in the discussion, with topical 0.03% tacrolimus as an active control [25]. In normal human epidermal keratinocytes (NHEK) and organotypic skin, osthol was compared with clobetasol propionate; selected analyses used 0.125 and 0.5 mg/mL [24]. These designs do not establish comparative efficacy of topical products at measured skin concentrations.

In the acute itch experiment, osthol was injected locally beneath the skin at the future pruritogen site 30 min before challenge. Blinded observers recorded scratching. Histamine-evoked scratching fell, whereas chloroquine-evoked scratching did not; neuronal calcium imaging and electrophysiology supported transient receptor potential vanilloid 1 (TRPV1)-associated modulation [23]. Local injection bypasses the stratum corneum and therefore provides no topical absorption estimate. The reported approximately 0.41 micromolar half-maximal value concerns histamine-evoked calcium influx, not clinical antipruritic potency.

Xanthotoxol acts at a different level of the itch pathway. Intraperitoneal or intrathecal dosing reduced acute itch and scratching in mouse models of dry skin and allergic contact dermatitis, with suppression of spinal gastrin-releasing peptide/receptor signaling [61]. The routes provide little information about topical efficacy or barrier repair. Its furanocoumarin structure also makes formulation-specific UVA testing necessary before skin use.

Umbelliferone reduced dermatitis phenotypes induced by DNCB and house-dust-mite extract in mice after oral dosing at 20 or 40 mg/kg and lowered inflammatory chemokines in stimulated HaCaT cells [30]. The test material was not authenticated as Apiaceae-derived. Isoscopoletin has only preliminary keratinocyte and basophil data [56]. In the scopoletin report, the mouse experiment used oral *Lycium barbarum* extract, while isolated scopoletin was tested only in keratinocytes [57]. A topical coumarin-derivative study included auraptene as a non-Apiaceae comparator and cannot establish an Apiaceae source [58].

*Peucedanum japonicum* Thunb. root extract reduced scratching and inflammatory readouts in mice and cell systems [79]. The result remains an extract-level finding because the study did not establish which constituent accounted for the response. It cannot be added to the single-coumarin evidence as if the administered materials were equivalent.

### 5.3. Imperatorin and Related Candidates for Psoriasis and Rosacea

Imperatorin reduced psoriasiform changes in imiquimod- and IL-23-driven mouse models [20]. In the imiquimod experiments, it was given by tail-vein injection at 1, 5, or 15 mg/kg in BALB/c mice and at 15 mg/kg in C57BL/6 mice, daily on days 3–6 after induction began on day 2. The IL-23 model used intraperitoneal imperatorin at 15 mg/kg on the days of cytokine injection. Both models used six mice per group and vehicle controls, without an established antipsoriatic drug comparator. These regimens test early intervention or co-treatment during induction. They provide systemic disease-model evidence but no topical absorption estimate or treatment evidence for established chronic lesions. Two induction systems in one report do not demonstrate independent replication.

Esculetin improved imiquimod-induced disease in a study involving regulatory T cells and nuclear factor kappa B (NF-kappaB) [31]. An esculetin-loaded lipid-carrier gel subsequently reduced inflammatory and histological readouts after UV irradiation of rat skin [21]. The latter study offers preliminary formulation evidence; uncertain UV-spectrum reporting and unmeasured skin exposure limit disease-specific interpretation. Neither study establishes efficacy of an Apiaceae preparation. A spectrum-effect study of *Angelica dahurica* identified six candidate coumarins and confirmed their anti-inflammatory activity in lipopolysaccharide (LPS)-stimulated RAW264.7 cells; phellopterin and isoimperatorin also reduced NF-kappaB-pathway readouts [80]. These cell-based results do not establish which constituents drive efficacy in skin or their concentrations in viable skin.

Wang and Zhu used commercial imperatorin at greater than 98% purity in an acute LL-37 model using BALB/c nude mice. The regimens included 20 mg/kg intraperitoneally before induction and a 3% ointment in vaseline applied twice daily for three days, with six mice per group and corresponding vehicle controls [27]. The topical regimen overlapped disease induction. Reduced redness, lesion area, and histological inflammation therefore support short-term model activity, without establishing treatment of persistent human rosacea.

The LL-37 study did not quantify imperatorin in viable skin or test the ointment under matched UVA exposure, and its efficacy groups did not include an active rosacea treatment. Ethanol was used to incorporate imperatorin into vaseline, while vaseline was described as the topical control; the final residual ethanol content was not specified. Confirmation should use a fully matched vehicle and treatment of established lesions before extending the conclusion to chronic disease.

### 5.4. Pigmentation-Modulating Candidates Outside Photochemotherapy

Osthol reduced melanin content and melanogenic-enzyme expression in B16F10 cells, with a reported half-maximal inhibition of 4.9 micromolar [81]. Umbelliferone was assessed in a four-week, double-blind, randomized split-face study involving 12 healthy Korean adults. Twice-daily 0.0025% ampoule application produced small changes in pigmentation measures; the reported four-week melanin-index reductions were 2.62% with umbelliferone and 0.03% with vehicle [28]. The clinical arm had no active comparator, although arbutin was used in cell assays. Clinical importance, persistence after withdrawal, and efficacy in diagnosed melasma or post-inflammatory hyperpigmentation remain unestablished.

### 5.5. Decursin-Class Compounds and Umbelliferone in Wound Repair

Decursin and decursinol angelate increased migration- and extracellular-matrix-related transcription in a HaCaT scratch assay [82]. That assay measures keratinocyte migration and cannot establish closure of a chronic wound. Systemic dosing also changes the chemical species under study. After oral decursin, rats were exposed mainly to decursinol [67]. Later rat and human studies of preparations containing decursin and decursinol angelate also found rapid loss of the parent compounds and decursinol-dominant systemic exposure [66,68]. A direct comparison of both parent compounds with decursinol at measured skin concentrations could identify the active species.

Umbelliferone was tested in high-glucose-exposed human dermal fibroblasts and diabetic mice with full-thickness excisional wounds. The methods specify oral administration at 10 mg/kg over a 10-week course, with dosing omitted on the wound-creation day and the following two days. Four 6-mm dorsal wounds were created per mouse and followed through day 18. The authors reported faster closure and changes in matrix, migration, and barrier-associated proteins [29]. This is in vivo wound evidence, not only a molecular or scratch-assay observation. Its limits are the experimental wound context, lack of measured tissue exposure and a standard-care adjunct comparison, and incomplete clarity about treatment timing across the methods and figure description.

### 5.6. Decursin-Class Compounds and Umbelliprenin in Melanoma

Decursin induced apoptosis in B16F10 cells and reduced tumor growth in C57BL/6 mice after intraperitoneal dosing [83]. Decursinol angelate was also reported to suppress melanoma-cell proliferation and tumor growth [84]. A correction identified duplication of a cyclin-dependent kinase 4 (CDK4) immunoblot with a cyclin-dependent kinase 2 (CDK2) panel and amended the corresponding immunoblot quantification and statistical analysis [84]. The authors stated that their conclusions were unchanged. The corrected study still comes from one group, and systemic exposure to the parent compound remains uncertain because of conversion to decursinol.

Umbelliprenin isolated from *Ferula szowitziana* inhibited human M4Beu melanoma cells through G1 arrest and caspase-dependent apoptosis [55]. That study provides cell-level activity without an animal prevention endpoint. The melanoma studies summarized here do not establish comparative benefit against current immunotherapy or targeted therapy, or identify the active compound and exposure in tumors. Replication in a specified molecular setting is needed before making treatment or prevention claims.

**Table 3:** Human and guideline evidence for PUVA and related photochemotherapy.

Treatment or Compound	Clinical Setting	Design and Route	Main Observation	Principal Limitation	Evidence Type	Relevance
Methoxsalen plus UVA	Psoriasis and other dermatoses	Clinical guidance; oral, bath, and topical protocols [9,75]	Drug and UVA are controlled together	Treatment burden and cumulative risk	Guideline and clinical treatment	Main PUVA reference; not a model for botanical use
Bergapten (5-MOP) plus UVA	Psoriasis and vitiligo	Clinical review and guideline context [9,10]	Therapeutic activity with a tolerability profile distinct from 8-MOP	Older, less extensive data and limited current availability	Human treatment and review	Selected clinical alternative within PUVA
Oral methoxsalen plus UVA	Psoriasis	Foundational clinical study [69]	Established therapeutic photochemotherapy	Historical protocol and comparator context	Human treatment	Supports the controlled drug-plus-UVA principle
Bath vs. systemic PUVA	Plaque psoriasis	Randomized, open, multicenter; 74 patients [73]	Both routes reduced PASI; the between-route difference was not significant	Open design and short follow-up	Comparative clinical trial	Route changes exposure and practical burden, not the core intervention
Oral vs. bath vs. cream 8-MOP	Healthy volunteers	Three-way crossover skin and plasma PK; 8 participants [72]	Topical routes increased cutaneous selectivity and reduced plasma exposure	Small PK study without an efficacy endpoint	Human pharmacokinetics	Direct basis for designing a skin-targeted protocol
PUVA vs. narrowband UVB	Vitiligo	Randomized double-blind comparison [45]	Narrowband UVB gave better color match and greater repigmentation after 48 treatment sessions; superiority in response durability was not established	Historical regimen and treatment era	Comparative clinical trial	Basis for the narrower contemporary use of PUVA
Bath-PUVA	Mycosis fungoides	Retrospective series; 62 patients [48]	Clinically meaningful response with a skin-directed route	No randomized comparator	Longitudinal human data	Selected role for local treatment
Repeated PUVA	Mycosis fungoides	Real-world cohort; 104 patients [47]	At least 250 sessions were associated with higher skin-cancer risk	Confounding by disease and treatment history	Longitudinal safety data	Cumulative-dose records and surveillance are part of care
PUVA	Plaque psoriasis with recent internal malignancy	Prospective cohort; 20 patients [78]	Mean PASI fell by about 69%; lower limbs responded less well	No comparator and a selected population	Prospective human cohort	Relevant to selected non-immunosuppressive treatment, not a general estimate
Bath or oral PUVA	Palmoplantar eczema or pustulosis	Controlled comparisons and guidance [39,40,41]	Activity reported, with inconsistent analyses and protocols	Small heterogeneous trials	Comparative trials and guidance	Possible option for selected patients
PUVA	Chronic prurigo	Swiss practice recommendations [36]	Listed as an option that may be considered	Weaker position than recommended therapies	Practice recommendations	Context for selecting an active comparator in pruritus
Khellin plus UVA	Vitiligo	Small early and open topical studies [59,62]	Repigmentation signals	Not a coumarin; weak design; hepatic signal [63]	Uncontrolled human data	Historical comparison only

5-MOP, 5-methoxypsoralen; 8-MOP, 8-methoxypsoralen; PASI, Psoriasis Area and Severity Index; PK, pharmacokinetics; PUVA, psoralen plus ultraviolet A; UVA, ultraviolet A. UVB, ultraviolet B.

**Table 4:** Disease-specific findings and study materials for non-PUVA compounds.

Compound or Material	Disease/Use and Model	Main Finding	Study Setting	Efficacy-Study Material	Main Limitation	Interpretation
Osthol	AD mice, keratinocytes, and organotypic skin	Related effects on the barrier, IL-31, H4R and TRP pathways [19,24,25]	Topical dosing during oxazolone induction (Hu et al. [19]); gavage in He et al. [25]; separate skin-culture experiments	Commercial osthol; Hu et al. specify Yuan Ye S26004 without purity; other studies have separate suppliers	Hu formulation and dose basis incompletely specified; no measured skin exposure-response or established-lesion regimen	Topical animal activity supports further local AD work; human biopsies provide association only
Osthol	Histamine itch mice and dorsal-root-ganglion neurons	Selective histamine-dependent antipruritic and TRPV1 effects [23]	Local subcutaneous pretreatment plus sensory-neuron assays	Commercial purified compound	Local injection bypasses the skin barrier; acute challenge rather than chronic-disease treatment	Supports sensory modulation; does not establish topical delivery
Osthol	Rabbit-skin microemulsion and edema model	Increased permeation and formulation-level anti-inflammatory readouts [26]	Excised rabbit skin; seven-day local irritation and edema observations	Purified osthol in a microemulsion system	Compared with an osthol-containing conventional gel; no viable human-skin retention or AD exposure-response	Formulation-level permeation and short local-tolerance evidence
Xanthotoxol	Acute, dry-skin, and contact-dermatitis itch models	Reduced scratching and spinal GRP/GRPR signaling [61]	Animal disease and spinal mechanism	Purified compound; botanical study-batch link not reported	Systemic or intrathecal dosing; UVA response not reported	Antipruritic activity only after non-topical administration
Umbelliferone	DNCB/DFE dermatitis mice and HaCaT cells	Reduced dermatitis and inflammatory chemokines [30]	Animal disease plus keratinocytes	Defined compound; an Apiaceae source was not reported	Oral route; no established local exposure-response or preparation-specific attribution	A separate topical disease study would test local relevance
Scopoletin	*Lycium* extract in mice; isolated compound in HaCaT cells	Animal and compound effects occurred in different materials [57]	Animal extract study plus cell assay	Non-Apiaceae extract and isolate	Scopoletin itself was not tested in the animal experiment	Mouse findings pertain to the extract, not isolated scopoletin
Isoscopoletin	Stimulated HaCaT and RBL-2H3 cells	Reduced cytokines and several signaling pathways [56]	Defined material tested in cell systems	Apiaceae source not reported	Only cultured cells were studied	A preliminary mechanistic observation
*Peucedanum japonicum* Thunb. root extract	Itch mouse model and immune-cell assays	Reduced scratching and inflammatory readouts [79]	Animal disease plus cell assays	Apiaceae root extract	Active constituent and exposure were not identified	The active constituent remains unidentified
Imperatorin	Neutrophils and psoriasiform mice	PDE4-linked suppression of neutrophil and disease phenotypes [20]	Tail-vein dosing in imiquimod mice; intraperitoneal dosing in IL-23 mice; six/group, vehicle controls	Isolated from *Angelica hirsutiflora*; stated purity >97%	Induction-period systemic intervention; no active antipsoriatic drug comparator, skin PK, or matched UVA test	Two induction systems in one report; topical psoriasis efficacy remains untested
Imperatorin	LL-37 rosacea-like mice and keratinocytes	Reduced inflammatory lesions with 20 mg/kg intraperitoneal pretreatment or a three-day 3% topical regimen [27]	LL-37 induction; six mice/group; vehicle controls	Commercial purified compound	Acute induction regimen; no active rosacea drug, measured viable-skin exposure, or UVA test	Short-term model activity; clinical treatment remains unestablished
Esculetin	Imiquimod psoriasis-like mice	Regulatory T-cell and NF-kappaB-related effects [31]	Animal disease	Defined compound; an Apiaceae source was not reported	One disease model; no measured skin exposure or preparation-specific Apiaceae link	A single preclinical comparison
Esculetin NLC gel	Short-term UV-irradiated rat skin lesions	F2 reduced TNF-alpha/CD34 and improved histology; F5 did not lower TNF-alpha significantly [21]	Formulation plus animal model	Commercial esculetin >98%; loaded F2/F5 gels	Free-drug comparator; no empty carrier. UV spectrum and dose basis unclear; no measured skin exposure	Exploratory formulation evidence; no clinical or Apiaceae-preparation efficacy
Decursin/decursinol angelate	HaCaT scratch assay	Increased wound-repair and matrix-related transcription [82]	Keratinocyte assay	Defined parent compounds	Keratinocyte assay; parent and metabolite must be compared in tissue	In vivo activity of either parent compound remains unknown
Umbelliferone	Full-thickness excisional wounds in diabetic mice; high-glucose fibroblasts	Faster experimental wound closure with matrix and barrier-related changes [29]	10 mg/kg orally; 10-week course reported; wound follow-up through day 18	Commercial umbelliferone from Sigma-Aldrich	No tissue PK or standard-care adjunct comparison; schedule and wound-level analysis need clarification	In vivo repair evidence requiring confirmation in a defined wound setting
Osthol	B16F10 melanogenesis assay	Reduced melanin and melanogenic-enzyme expression [81]	Cell assay	Defined compound in a *Cnidium monnieri* (L.) Cusson-linked study	No controlled human pigmentation study	Cell-level depigmentation finding without human data
Umbelliferone	Melanogenesis assays; four-week split-face study in 12 healthy adults	Small pigmentation-index changes with 0.0025% ampoule [28]	Cells plus double-blind vehicle-controlled human formulation study	Commercial compound; identical containers and light-protective packaging	No clinical active comparator or diagnosed disease cohort; short follow-up	Preliminary controlled human result awaiting independent confirmation
Decursin	B16F10 cells and tumor-bearing mice	Apoptosis and reduced tumor growth [83]	Cells plus animal tumor model	Defined compound	PK studies show metabolite-dominant systemic exposure	Preliminary antitumor activity with uncertain active species
Decursinol angelate	Melanoma cells and tumor-bearing mice	Apoptosis and reduced tumor growth [84]	Cells plus animal tumor model	Defined compound	One corrected study and the parent-metabolite problem	Corrected report; independent confirmation remains necessary
Umbelliprenin	Human metastatic melanoma cells	G1 arrest and caspase-dependent apoptosis [55]	Cell assay	Isolated from *Ferula szowitziana* DC.	No animal exposure or prevention endpoint	Activity reported only in cultured cells

AD, atopic dermatitis; DFE, *Dermatophagoides farinae* extract; DNCB, 2,4-dinitrochlorobenzene; GRP/GRPR, gastrin-releasing peptide/receptor; H4R, histamine H4 receptor; NLC, nanostructured lipid carrier; PDE4, phosphodiesterase 4; PK, pharmacokinetics; TRP, transient receptor potential; UVA, ultraviolet A. IL, interleukin; LL-37, human cathelicidin peptide; NF-kappaB, nuclear factor kappa B; TNF, tumor necrosis factor; TRPV1, transient receptor potential vanilloid 1; UV, ultraviolet.

### 5.7. Mechanistic Evidence and Causal Interpretation

Changes in pathway markers require different interpretation from functional perturbation. Zonula occludens-3 (ZO-3) knockdown lowered transepithelial resistance in HaCaT cells, and osthol attenuated SC79-associated p-Akt signals and ZO-3 loss [19]. However, the study did not test whether ZO-3 knockdown abolished osthol efficacy or whether osthol restored transepithelial resistance. Its three-patient skin-biopsy comparison was observational. Calcium imaging and electrophysiology support modulation of histamine-responsive sensory signaling, while cytokine and receptor-expression assays do not identify a direct molecular target [23,24]. Peroxisome proliferator-activated receptor (PPAR) antagonists implicated that pathway in keratinocyte IL-31RA regulation [25]. These experiments do not establish one continuous barrier-to-neuron mechanism.

For imperatorin, purified-enzyme assays showed preferential inhibition of phosphodiesterase 4 (PDE4) subtypes PDE4A, PDE4B, and PDE4C among the tested subtypes. The protein kinase A (PKA) inhibitors H89 and KT5720 reversed selected responses in activated human neutrophils [20]. This supports PDE4/cyclic adenosine monophosphate (cAMP)/PKA involvement in the cellular effects, but the dermatitis experiments did not establish target dependence in vivo, and off-target activity remains possible. The rosacea study combined c-Jun N-terminal kinase 1 (JNK1) knockdown, co-immunoprecipitation, and a cellular thermal-shift assay with docking [27]. These experiments support JNK1/signal transducer and activator of transcription 1 (STAT1) pathway involvement and altered protein association; they do not establish a unique binding site, binding affinity, or that direct engagement of both proteins is necessary for the animal response.

Decursin and decursinol angelate pose a different attribution problem: the pharmacokinetic studies demonstrate substantial conversion to decursinol, while the skin and tumor experiments describe responses to administered parent compounds [66,67,68,82,83,84]. Paired tissue measurements and direct parent-metabolite comparisons are needed to identify the active species.

## 6. Formulation, Skin Exposure, and Metabolism

The route of methoxsalen administration affects both exposure and risk during PUVA. Oral methoxsalen causes systemic photosensitivity, which necessitates eye protection and precautions against sunlight. Bath or local PUVA lowers plasma exposure and concentrates the drug in skin [72]. Even so, uneven psoralen distribution can cause localized phototoxic burns, and photosensitivity may persist after treatment [9,74].

The osthol microemulsion gel was evaluated for permeation across excised rabbit skin and for local irritation and formalin-associated edema in rabbits. Its comparison gel also contained osthol, in a phosphate buffer solution (PBS)/Carbomer base, and local observations extended over seven days [26]. The findings support a formulation effect under those conditions. They do not establish drug retention in viable human skin, an atopic-dermatitis exposure-response relationship, or longer-term tolerance. Increased passage into a diffusion-cell receiver should not be treated as measured systemic bioavailability.

Khalil et al. [21] compared free esculetin suspension with two loaded nanostructured lipid carrier (NLC) gels after UV irradiation of rat skin. F2 reduced TNF-alpha and CD34, whereas F5 did not significantly lower TNF-alpha. The reported groups included no empty-carrier or active-drug arm. The study measured dialysis-membrane release, not skin permeation or tissue drug levels. Its UV-B designation conflicts with the reported 254-nm wavelength, and the animal dose labels (1%/2%) are not reconciled with the 0.1% *w*/*v* formulation loading. These limitations prevent attribution of the effect to enhanced skin delivery or extrapolation to clinical psoriasis.

Rapid first-pass metabolism converts decursin and decursinol angelate to decursinol. In rats given oral decursin, only decursinol was detected in plasma, and little parent compound escaped hepatic elimination [67]. Later rat and human studies likewise found decursinol-dominant systemic exposure after administration of preparations containing the parent compounds [66,68]. It is not known whether decursinol reproduces the desired skin activity or whether local delivery can preserve the parent compounds. Simultaneous measurement of the parent compounds and decursinol would clarify exposure under each delivery route; their skin activity would also need to be compared.

Formulation evidence also includes chemical stability. In the umbelliferone ampoule study, approximately 73% of the starting compound remained after four weeks of sunlight exposure, whereas storage at the tested temperatures had less effect [28]. This supports attention to light-protective packaging and photoproducts; it is not a photoirritation test. Across candidates, stability, release, viable-skin distribution, plasma exposure, and repeated-use tolerance should be linked to the same formulation used for efficacy testing.

## 7. Photosafety and Product Control

### 7.1. Acute Injury, Photoallergy, and Cumulative Carcinogenicity Are Distinct

Phytophotodermatitis follows skin contact with a phototoxic plant or product and subsequent light exposure. It can cause burning, erythema, vesicles, or bullae, followed by delayed hyperpigmentation [85]. Risk varies with species, organ, plant stress, preparation, constituent content, vehicle, skin hydration, and UVA dose. Differences in *Heracleum* metabolomic profiles also caution against assuming that every species has the same photosensitizing potential [86].

An occupational episode of phytophotodermatitis affected 11 celery harvest workers [87]. It was associated with high psoralen content in the crop, clear weather, moist hands, inadequate protection, and individual skin susceptibility. A recent clinical report also distinguished ordinary dietary consumption from repeated handling or unusually high exposure [88]. Prevention depends on both the composition of the material and how it is handled.

Photoallergy differs immunologically from direct phototoxicity. In one plant-contact case, photopatch reactions were positive to 8-MOP, 5-MOP, and imperatorin but negative to trimethylpsoralen [89]. Findings from one sensitized patient cannot establish relative phototoxic potency. The positive reactions indicate that allergic photocontact reactions should be considered after plant or product exposure.

Cumulative carcinogenicity after PUVA is a third endpoint. The long-term psoriasis cohort and the mycosis-fungoides observations concern repeated therapeutic exposure [47,76]. Acute photoirritation and sporadic plant contact involve different doses and timescales. A negative short-term photoirritation result consequently cannot exclude delayed genotoxic or carcinogenic effects.

### 7.2. Assessment of Formulation-Specific Photosafety

The 3T3 neutral-red-uptake assay is useful as an initial phototoxicity screen, especially for identifying negative materials, although positive results can overpredict clinical risk [90]. Lipophilic coumarins may precipitate or remain poorly soluble, distorting concentration-response curves. A modified protocol for poorly water-soluble materials addresses part of this problem [91]. Reconstructed human skin can then provide information on tissue bioavailability and the barrier. When assays disagree, a staged follow-up is more informative than a binary safe-or-unsafe label.

Initial testing of a topical coumarin or botanical product should include ultraviolet-visible absorption, photostability, and a cell assay suited to the material’s solubility. Reconstructed human skin and chemical analysis before and after irradiation add information on tissue exposure and photoproducts. Photogenotoxicity and repeated-use studies become relevant as exposure increases. The dose- and UVA-dependent photoirritant and photogenotoxic effects summarized in the furanocoumarin literature [13] motivate this proposed testing sequence, but safety must be assessed for the finished product.

### 7.3. Food, Cosmetic, Supplement, and Medicinal Products Need Different Controls

Analytical surveys have detected furanocoumarins in Apiaceae vegetables and herbs, with concentrations that vary by species, plant organ, storage, and processing [92]. Ordinary dietary exposure differs from PUVA in dose, route, and control [93]. Exposure may increase with concentrated supplements, unusually high intake, or infected or stressed plants. Metabolic interactions and simultaneous dermal and solar exposure also affect risk. A systematic review found moderate evidence of an association between higher dietary furanocoumarin intake and melanoma risk, while heterogeneity and limitations in dietary and UV-exposure assessment prevented a definitive causal conclusion [94]. Risk assessment requires compound-level measurements and a documented exposure history.

Cosmetics place the material directly on skin that may receive sunlight. Annex II, entry 358 of the European Union (EU) Cosmetics Regulation specifies that furocoumarins must be below 1 mg/kg in sun-protection and bronzing products [95]. A high-performance liquid chromatography–tandem mass spectrometry (HPLC-MS/MS) method has been developed to quantify trace furocoumarins in finished cosmetics [96]. Studies of bergamot oil and other essential oils show that composition, concentration, solvent, and UVA jointly determine the hazard [97,98,99]. The finished formulation must be tested under realistic conditions; the source plant, ingredient name, and dark cytotoxicity are not adequate substitutes.

Supplements add uncertainty about both identity and drug interactions. In a 2025 survey of ten *Heracleum sphondylium* L. supplements, seven had no detectable target furanocoumarins, one was near a proposed threshold, and two substantially exceeded it [100]. The plant name on the label did not predict exposure. Furanocoumarins can inhibit cytochrome P450 (CYP)-mediated metabolism and affect P-glycoprotein transport; uptake-transporter effects described for fruit juices may also involve co-occurring flavonoids [101]. Coumarin safety varies markedly with structure and dose [102]. Medication history should be documented for dermatology patients taking retinoids, immunomodulators, antihistamines, or other photosensitizing drugs.

Traditional pigmentation treatments need particular scrutiny when users intentionally add light exposure. In a systematic review of medicinal plants for vitiligo or hypermelanosis, reporting of phototoxic, gastrointestinal, hepatic, and other adverse events was incomplete [103]. Both the light protocol and adverse events need to be reported alongside efficacy.

## 8. Discussion

### 8.1. Relating Traditional Use to Experimental Evidence

The clearest connection between traditional skin use and experimental pharmacology in this collection is material-specific. In the *Cnidium monnieri* study, a 70% ethanol extract of authenticated fruit and osthol isolated from the same material were evaluated side by side. Both oral preparations reduced contact-hypersensitivity ear swelling, providing a traceable link between the plant material, one constituent, and a selected inflammatory outcome [22]. The extract also reduced scratching, but that experiment did not assign the antipruritic effect to isolated osthol. The result therefore gives partial biological support to the traditional concern with pruritic, inflammatory lesions without establishing equivalence to traditional preparations or topical efficacy. The study also used an oral hydroalcoholic extract rather than the external aqueous preparation recorded in the pharmacopoeia (Section 4.1). This comparison anchors further investigation of inflammation, barrier function, and itch to a specified botanical material, while leaving the contribution of osthol to each function to be tested.

*Angelica* illustrates why botanical source and indication alignment must be considered separately. A review describes external use of *A. dahurica* root for boils, carbuncles, sores, and painful swellings, which provides historical context for testing inflammatory skin effects [52]. The modern imperatorin evidence instead comes from a compound isolated from *A. hirsutiflora* in systemic psoriasis models and from commercial imperatorin in a short topical rosacea-like experiment [20,27]. These studies remain informative at the compound level, but they do not establish equivalence to that traditional root preparation or its uses. Interpretation of traditional use should retain the species, organ, preparation, indication, and route, whereas purified imperatorin can be assessed independently through the relevance of its disease model, dose, route, and comparator. The two paths are compatible but answer different questions.

### 8.2. Interpreting Mechanisms Through Skin Exposure and Light Response

Mechanistic studies refine the biological meaning of symptom-level links by separating inflammation, barrier function, and sensory itch. Osthol altered dermatitis-associated signals and ZO-3 expression, while other experiments supported effects on histamine-responsive sensory signaling [19,23]. These complementary findings justify assessing the functions together, but they do not form a single causal barrier-to-neuron pathway. The Hu study did not establish that ZO-3 was necessary for osthol’s effect or demonstrate restoration of transepithelial resistance by osthol; local injection before an acute itch challenge bypassed the stratum corneum. Oral dosing, local injection, and culture concentrations also represent different exposures [19,23,25]. A prospective topical study should therefore use one defined formulation and measure inflammation, scratching, and functional barrier recovery while relating these outcomes to measured skin concentrations.

PUVA provides the clearest example of why the administered material cannot be interpreted apart from exposure and light. The established intervention is a defined photosensitizer delivered by a specified route and paired with a controlled UVA protocol. Bath or local methoxsalen can concentrate exposure in skin while lowering plasma exposure [72]. Psoralen distribution and application-to-irradiation timing still require control to limit phototoxic burns, and photosensitivity may persist after treatment [9,74]. Repeated therapeutic exposure also carries a cumulative skin-cancer association that differs from acute plant contact [76]. PUVA therefore establishes a clinical role for controlled drug–UVA regimens, not for psoralen-containing plants or other furanocoumarins as interchangeable treatments. For candidates intended as non-phototherapy topicals, ambient light is part of the intervention context, and each finished formulation needs its own light-response margin at the intended exposure.

Formulation and metabolism further determine which intervention reaches the tissue. Oral decursin and decursinol angelate produce decursinol-dominant systemic exposure, but this conversion does not identify the active chemical species in skin [67,68]. Likewise, greater passage of an osthol microemulsion into the receiver compartment across excised rabbit skin does not demonstrate retention in viable human epidermis or dermis [26]. Such gaps affect both efficacy attribution and safety interpretation: the administered parent compound, tissue-exposed species, and constituent in the original preparation may differ. Efficacy, parent–metabolite measurements, and light response should therefore be assessed under the same formulation and dosing conditions. A botanical product additionally requires a reproducible compositional profile, while a purified compound requires defined identity, purity, and formulation rather than source-based assumptions.

### 8.3. Implications for Further Development

These distinctions place osthol and imperatorin at different decision points rather than on a single evidence ranking. For osthol, the *Cnidium* material link and complementary dermatitis, barrier-associated, and sensory findings make a topical test of established lesions informative. The key question is whether one defined formulation can produce reproducible local exposure and improve disease-relevant functions against an appropriate comparator; the topical Hu experiment overlapped disease induction and did not measure skin concentrations [19]. Imperatorin requires a different separation of claims. The two psoriasis induction systems came from one report, while the rosacea study addressed a different disease and regimen; together they do not establish replicated topical efficacy in either indication [20,27]. Its next step depends on replicated local benefit with a fully matched vehicle and an acceptable light-response margin for the same formulation. Table 5 records the corresponding study designs.

Apparent proximity to translation does not make dermatological endpoints interchangeable. Vehicle-controlled application of umbelliferone in 12 healthy adults provides a human observation on pigmentation, while leaving efficacy and durability in diagnosed pigmentary disease unresolved [28]. Oral umbelliferone in diabetic mice provides an in vivo wound-closure result, but its route and experimental excision setting do not establish topical benefit for chronic human ulcers [29]. Each finding advances a distinct question; together they do not support a general skin-efficacy claim or validate an Apiaceae preparation. The next study should follow the function required by the intended indication, with a relevant comparator and a matched material and route. The presence of human observations or a larger number of pathway measurements cannot replace that correspondence.

**Figure 3:**
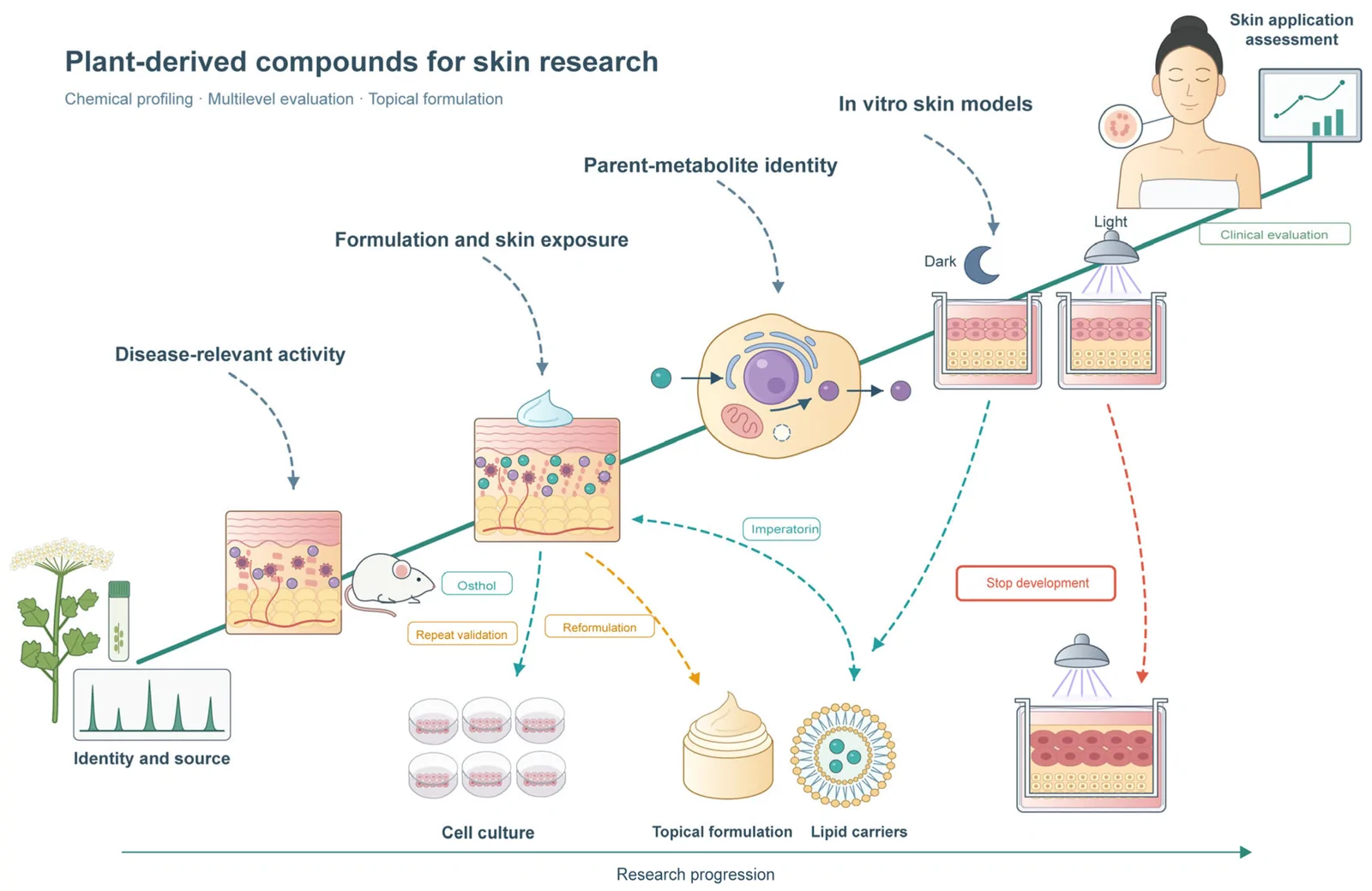
Proposed workflow for dermatologic development of plant-derived compounds. Identity and source authentication precede disease-relevant testing, formulation and skin-exposure studies, parent-metabolite assessment, and evaluation in skin models under matched dark and light conditions. Osthol and imperatorin illustrate preclinical candidates requiring further validation. The branches indicate repeat validation, reformulation, or discontinuation when findings are irreproducible, delivery is inadequate, or phototoxicity is unacceptable. Clinical evaluation is a prospective stage for non-PUVA candidates; established PUVA protocols require continued monitoring of cumulative UVA exposure. OpenAI GPT (see Section 2.5) assisted with preparation of this proposed development workflow and its labels. The illustration presents prospective testing and decision stages. The authors reviewed the AI-assisted content; the illustration was redrawn and finalized in Adobe Illustrator. It contains no primary experimental images or measurements. PUVA, psoralen plus ultraviolet A; UVA, ultraviolet A.

The resulting priorities are conditional because this narrative review compared heterogeneous diseases, materials, routes, and outcomes through selective retrieval. The main texts of the eleven primary studies assembled as described in Section 2.3 were inspected in detail, but the full reference collection was not re-assessed in full, and the independent Supplementary Files for Hu, Tsai, and Khalil were not inspected. No exhaustive search or formal risk-of-bias analysis was performed. Figure 2 therefore maps evidence coverage rather than clinical readiness or comparative efficacy. For traditional products, the informative design is a traceable comparison of a defined extract and its constituents against matched symptoms and functions. For purified compounds, the central question is efficacy at a relevant indication, route, tissue exposure, and light condition. Figure 3 and Table 5 translate these distinct aims into a shared sequence of material verification, disease-relevant testing, formulation, exposure, and safety assessment.

## 9. Conclusions

Dermatological evidence can inform ethnopharmacological interpretation when the relationship between the tested material and traditional use is explicit. The authenticated *Cnidium monnieri* fruit extract and same-material osthol comparison provide a partial biological link for selected inflammatory outcomes, while leaving the traditional preparation and topical efficacy unresolved. Defined psoralen–UVA therapy answers a different question: it is an established clinical intervention whose use and risk are determined by the photosensitizer, route, UVA protocol, and cumulative exposure, rather than by botanical occurrence alone.

Osthol and imperatorin remain conditional priorities for preclinical work because their existing findings identify different, testable gaps. Progress requires disease-relevant functional endpoints, reproducible material and formulation, exposure in the target tissue, and light-response testing under the same conditions. These requirements also prevent healthy-volunteer pigmentation, experimental wound closure, or pathway changes from being treated as general skin efficacy. Activity of an isolated constituent does not by itself validate the traditional use of its source plant. Studies that compare the traditional preparation, a chemically characterized extract, and its constituents against the same skin function can test that link directly, whereas compound-level studies define which modern interventions warrant further development.

## Figures and Tables

**Table 2:** Plant sources, study materials and dermatological evidence for the principal compounds.

Compound or Group	Structural Class	Documented Apiaceae Occurrence	Material Used in Main Efficacy Studies	Dermatology Evidence	Main Limitation
Methoxsalen (xanthotoxin, 8-MOP)	Linear furanocoumarin	Detected and quantified in authenticated *Levisticum officinale* roots [60]	Defined pharmaceutical photosensitizer	Human results apply to a controlled drug-plus-UVA protocol [7,9]	Main clinical reference for PUVA
Bergapten (5-MOP)	Methoxylated linear furanocoumarin	Occurs in Apiaceae [49]	Defined clinical photosensitizer	Human treatment and comparative data [9,10]	Selected PUVA alternative; the data do not describe a plant extract
Imperatorin	Prenyloxy linear furanocoumarin	Reported in *Angelica dahurica* (Hoffm.) Benth. & Hook.f. ex Franch. & Sav. [52,53]	Isolated from *Angelica hirsutiflora*, purity >97%, in the psoriasis study; commercial material in the rosacea study	Psoriasis- and rosacea-like models [20,27]	Relationship among topical activity, skin exposure, and UVA response is unknown
Xanthotoxol	Hydroxy linear furanocoumarin	Reported in *Angelica dahurica* [52]	Purified compound not linked to an Apiaceae study batch	Systemic and intrathecal antipruritic data [61]	No topical exposure-response or UVA study
Osthol	Prenylated simple coumarin	Isolated from *Cnidium monnieri* (L.) Cusson [22]	Osthol isolated from authenticated fruit in the extract–constituent comparison [22]; commercial purified material in later studies [19,23]	Findings from itch, atopic-dermatitis, barrier and skin systems	Viable-human-skin exposure and clinical efficacy remain unestablished
Umbelliferone	Hydroxy simple coumarin	Detected in authenticated *Levisticum officinale* roots by LC–MS [60]	Commercial compound (Sigma-Aldrich, St. Louis, MO, USA, as identified in the wound and pigmentation reports)	Oral dermatitis and diabetic-wound findings; topical pigmentation measurements in healthy volunteers [28,29,30]	The botanical source is separate from compound efficacy; clinical disease benefit remains unresolved
Scopoletin and isoscopoletin	Methoxylated simple coumarins	Scopoletin reported in *A. dahurica* [52]; isoscopoletin reported in *Ammi majus* [56]	Non-Apiaceae isolate or unresolved defined material	Extract, keratinocyte and basophil observations [56,57]	Discovery and mechanistic work only
Esculetin	Catechol-type simple coumarin	Detected in authenticated *Levisticum officinale* roots by LC–MS [60]	Defined compound or formulation not linked to an Apiaceae batch	Psoriasis-like and formulation studies [21,31]	Limited model replication and exposure data; no preparation-specific Apiaceae efficacy claim
Decursin and decursinol angelate	Dihydropyranocoumarin esters	Major *Angelica gigas* Nakai constituents [54]	Defined parent compounds from sources that varied among studies	Wound and melanoma findings are complicated by conversion to decursinol	Active parent or metabolite in tissue remains unresolved
Umbelliprenin	Sesquiterpene coumarin	Isolated from *Ferula szowitziana* DC.	Study material was isolated from *Ferula szowitziana* [55]	Melanoma-cell data only	Exposure and efficacy have not been tested in vivo
Khellin	Furanochromone, not a coumarin	Occurs in *Visnaga daucoides* Gaertn. (syn. *Ammi visnaga* (L.) Lam.)	Defined khellin or liposomal khellin	Historical UVA-assisted vitiligo evidence with hepatic and design limitations [59,62,63]	Historical non-coumarin comparator

5-MOP, 5-methoxypsoralen; 8-MOP, 8-methoxypsoralen; PUVA, psoralen plus ultraviolet A; UVA, ultraviolet A; LC–MS, liquid chromatography–mass spectrometry. Khellin is an Apiaceae-derived furanochromone and is chemically distinct from coumarins. Studies of khellin with UVA are included only for historical comparison.

**Table 5:** Evidence-based next studies for Apiaceae-associated treatments and compounds.

Disease Setting	Current Apiaceae Evidence	Candidate or Intervention	Main Unresolved Problem	Next Study	Current Status
Atopic dermatitis and chronic pruritus	Preclinical only outside PUVA	Osthol; xanthotoxol and umbelliferone as secondary leads	Topical induction-period activity plus oral, injection, and culture results; no independently replicated defined topical product [19,23,25]	Replicate a defined topical osthol formulation after lesions are established, with active comparator, skin PK, barrier, itch, inflammation, and tolerance	Conditional priority based on complementary models, not comparative efficacy
Psoriasis	Established PUVA; preclinical non-PUVA evidence	Methoxsalen/PUVA; imperatorin	PUVA burden and cumulative risk; imperatorin skin exposure and UVA margin are unknown [20,76]	Lower-exposure PUVA studies in selected patients; topical imperatorin against current care under matched dark and UVA conditions	PUVA is established; systemic imperatorin models do not establish topical psoriasis efficacy
Palmoplantar inflammatory disorders	Selected PUVA option	Methoxsalen/PUVA	Small heterogeneous studies and uncertain schedule [39,40,41]	Disease-specific comparative trial with a prespecified route and schedule	Evidence cannot be extrapolated from plaque psoriasis
Rosacea	Preclinical only	Imperatorin	Acute induction model, no active comparator or measured skin exposure/UVA response [27]	Treat established inflammatory lesions with a fully matched vehicle and active comparator; measure exposure and UVA response	Short topical model signal; priority depends on confirmation
Vitiligo	Selected historical PUVA use; no non-PUVA lead	Methoxsalen- or bergapten-PUVA	Narrowband UVB and current topical treatments provide a better benchmark for most patients [4,44,45]	Indication-specific comparison only where PUVA remains clinically justified	Non-PUVA pigment-reduction findings do not support a vitiligo efficacy claim
Hyperpigmentation	Preliminary formulation evidence	Umbelliferone; osthol at cell level	Healthy-volunteer study; clinical importance and persistence unresolved [28]	Larger controlled trial in a named disorder with colorimetry, durability, irritation, and light testing	Umbelliferone requires independent confirmation
Impaired wound repair	Umbelliferone mouse wound closure; decursin-class keratinocyte data	Umbelliferone; decursin-class compounds	No tissue exposure or standard-care adjunct comparison; parent-metabolite and schedule questions differ [29,82]	Confirm a defined diabetic-wound regimen with animal-level analysis and tissue exposure before clinical testing	Umbelliferone merits confirmatory preclinical work; human-ulcer benefit remains unestablished
Melanoma	Discovery evidence only	Decursin, decursinol angelate, and umbelliprenin	Limited replication, uncertain active species, and no clinically relevant comparator [55,83,84]	Molecularly defined in vivo model with PK, independent replication, and current-treatment comparator	No therapeutic or prevention claim is supported
Early mycosis fungoides	Established selected PUVA use	Methoxsalen/PUVA	Response duration, treatment burden, and cumulative cancer risk [47,48]	Skin-directed protocols with long-term response and surveillance data	Controlled PUVA remains clinically relevant
Product safety	Exposure-control problem	Furanocoumarin-rich extracts and finished products	Composition, dose, UVA exposure, and interactions vary [13,100]	Analyte-resolved testing of the finished product under realistic use	Safety cannot be inferred from the plant or compound class

PK, pharmacokinetics; PUVA, psoralen plus ultraviolet A; UVA, ultraviolet A. Each row refers to the stated disease, route, and material. UVB, ultraviolet B.

## Data Availability

No new experimental datasets were generated. Literature-derived tables, nomenclature records, the domain-specific Figure 2 source map, and the selected study-level evidence table are supplied with the article. The underlying publications are identified in the references.

## Supplementary Materials

The following supporting information can be downloaded at: https://www.mdpi.com/article/10.3390/molecules31213996/s1, Table S1: Selected traditional uses and tested-material links; Table S2: Botanical nomenclature records; Table S3: Qualitative source map for Figure 2; Table S4: Study-level evidence underlying candidate interpretation; Table S5: Record of literature searches and evidence acquisition; comma-separated values (CSV) files accompanying Tables S3 and S4: domain-specific references and qualifications for Figure 2, and the study-level evidence extraction, respectively. The supplementary tables cite the sources in [7,9,11,19,20,21,22,23,24,25,26,27,28,29,30,31,50,51,52,53,54,55,56,57,59,60,61,62,63,66,67,68,69,72,73,76,79,81,82,83,84,89]; botanical nomenclature records are provided in [104,105,106,107,108,109,110,111,112,113,114].

## Abbreviations

The following abbreviations are used in this manuscript:

5-MOP	5-methoxypsoralen
8-MOP	8-methoxypsoralen
AD	atopic dermatitis
CDK	cyclin-dependent kinase
DFE	*Dermatophagoides farinae* extract
DNCB	2,4-dinitrochlorobenzene
GBD	Global Burden of Disease
GRP/GRPR	gastrin-releasing peptide/receptor
H4R	histamine H4 receptor
IL	interleukin
IWGDF	International Working Group on the Diabetic Foot
JAK	Janus kinase
NLC	nanostructured lipid carrier
NF-kappaB	nuclear factor kappa B
PASI	Psoriasis Area and Severity Index
PDE4	phosphodiesterase 4
PK	pharmacokinetics
PPAR	peroxisome proliferator-activated receptor
PUVA	psoralen plus ultraviolet A
TNF	tumor necrosis factor
TRP	transient receptor potential
UVA	ultraviolet A
UVB	ultraviolet B
