# Supplementary material for: Dermatological Evidence for Coumarins Occurring in Apiaceae: Separating Traditional Use, Botanical Occurrence, and Compound Pharmacology
Source: Molecules. 2026 Nov 5;31(21):3996. doi: 10.3390/molecules31213996 (PMC13764724; doi:10.3390/molecules31213996)
Supplement: Supplementary file 1 [file molecules-31-03996-s001.zip › Supplementary_Materials_S1-S5_revised.pdf]

Supplementary information

Dermatological evidence for coumarins occurring in Apiaceae: separating traditional use, botanical occurrence, and compound pharmacology

Table S1 Selected traditional uses and tested-material links

Material	Traditional-use basis	Tested material and experiment	Permitted interpretation	Evidence level (Section 2.4)
Cnidium monnieri (L.) Cusson fruit (Cnidii Fructus)	Chinese Pharmacopoeia (2020): indications include eczema and pruritus; external use mostly as a decoction for fumigation and washing, or as a powder made into a paste; minimum osthol content 1.0% in the dried fruit. Use for pruritic/suppurative dermatitis is also described by Matsuda et al. (2002).	Voucher specimen CM-99001, deposited at Kinki University (now Kindai University), Osaka; 70% ethanol fruit extract and osthol isolated from that material; oral mouse contact-hypersensitivity models.	Extract and osthol reduced ear swelling. The scratching endpoint belongs to the extract arm. The oral hydroalcoholic extract differs from the external aqueous preparation; no topical clinical validation.	Documented traditional use (pharmacopoeia); extract-level activity and isolated-compound pharmacology from the same authenticated material.
Angelica dahurica (Hoffm.) Benth. & Hook.f. ex Franch. & Sav. root (Angelicae Dahuricae Radix)	External use for boils, carbuncles, sores, and painful swellings, summarized from pharmacopoeial sources by a review (Zhao et al., 2022). Chinese Pharmacopoeia (2020): indications include sores and painful swellings; minimum imperatorin content 0.080%; no topical preparation described.	Secondary traditional-use account; commercial imperatorin was used in the rosacea model (Wang and Zhu, 2026).	Provides context, but does not equate traditional preparations or indications with a purified-compound rosacea treatment.	Documented traditional use (secondary review and pharmacopoeia); botanical occurrence of imperatorin; isolated-compound pharmacology (commercial imperatorin).
Ferula szowitziana DC.	No traditional melanoma-treatment claim is made here	Umbelliprenin isolated from the plant used in the melanoma-cell study (Barthomeuf et al., 2008)	Source-linked compound discovery; no inference of traditional melanoma efficacy.	Botanical occurrence (isolation); isolated-compound pharmacology in cells. No documented traditional use assessed.
Peucedanum japonicum Thunb. root extract	No preparation-specific traditional antipruritic claim is established by this review	Root extract tested in an itch model (Kim et al., 2025)	Activity remains at the extract level; the active coumarin was not identified.	Extract-level activity. No documented traditional use assessed.

Material	Traditional-use basis	Tested material and experiment	Permitted interpretation	Evidence level (Section 2.4)
Ammi majus L. fruit	Powdered fruit taken orally before sun exposure for leukoderma in Egyptian folk medicine (Pathak and Fitzpatrick, 1992).	Methoxsalen (8-MOP) and related furanocoumarins isolated from the fruit in the 1940s; subsequent clinical use as a defined drug with controlled UVA (PUVA).	Clinical PUVA evidence applies to the defined drug–UVA regimen, not to the crude fruit preparation, in which psoralen content and light exposure were not standardized.	Documented traditional use (historical review); isolated-compound pharmacology with clinical evidence for the defined drug only.

These are selected links used to interpret the review, not an exhaustive ethnobotanical inventory. Primary experimental evidence and secondary descriptions of traditional use are identified separately. The evidence-level column applies the four levels defined in Section 2.4 of the main text; a finding at one level is not taken as evidence at another.

Table S2 Botanical nomenclature records

Access date: 5 September 2026 unless otherwise stated. Names below reflect the specified WFO or POWO records. Source-specific names and alternative spellings are retained where needed to identify the published material. Nomenclatural records do not authenticate an experimental plant organ, voucher, or batch.

Botanical name	Database record	Nomenclature note
Cnidium monnieri (L.) Cusson	https://powo.science.kew.org/taxon/urn:lsid:ipni.org:names:840463-1; https://www.worldfloraonline.org/taxon/wfo-0000612807	Accepted name in POWO, with basionym *Selinum monnieri* L.; the Chinese Pharmacopoeia also uses the parenthetical author. The WFO record lists the name without the parenthetical author. POWO accessed 30 October 2026.
Angelica dahurica (Hoffm.) Benth. & Hook.f. ex Franch. & Sav.	https://www.worldfloraonline.org/taxon/wfo-0000536154	WFO full author string.
Angelica gigas Nakai	https://www.worldfloraonline.org/taxon/wfo-0000536198	WFO name record.
Peucedanum japonicum Thunb.	https://www.worldfloraonline.org/taxon/wfo-0001068875	WFO name record.
Ferula szowitziana DC.	https://www.worldfloraonline.org/taxon/wfo-4000014698	Spelling listed by WFO; source study and current POWO use *Ferula szowitsiana*. Both spellings retained in the first occurrence.
Lycium barbarum L.	https://www.worldfloraonline.org/search?query=Lycium+barbarum	WFO accepted-name search result; exclude Lam. homonym.
Visnaga daucoides Gaertn. (syn. *Ammi visnaga* (L.) Lam.)	https://wfoplantlist.org/taxon/wfo-0000421889-2026-06	Accepted as *Visnaga daucoides* Gaertn. (wfo-0000421889) in the June 2026 WFO Plant List. *Ammi visnaga* (L.) Lam. (wfo-0000531177) is a synonym retained to identify the published material. Accessed 5 September 2026.
Heracleum sphondylium L.	https://www.worldfloraonline.org/taxon/wfo-0000720220	WFO name record.

Botanical name	Database record	Nomenclature note
Angelica hirsutiflora Tang S.Liu, C.Y.Chao & T.I.Chuang	https://powo.science.kew.org/taxon/urn:lsid:ipni.org:names:837648-1	Accepted species in POWO. The name reported by Tsai et al. (2021) is retained; this record does not verify the tested botanical batch.
Levisticum officinale W.D.J.Koch	https://powo.science.kew.org/taxon/844187-1	Accepted species in POWO, Apiaceae; accessed 30 September 2026. Nomenclature record only; botanical authentication of the roots is reported separately in Olennikov (2023).

Table S3 Qualitative source map for Figure 2

The accompanying Table_S3_Figure2_domain_sources.csv contains one row per material and evidence domain, with domain-specific citations, current main-manuscript reference numbers, and qualifications. Direct and limited describe evidence coverage, not quality or clinical readiness. Empty citation fields in unestablished cells indicate no qualifying study in this selected collection, not proof of absence. Triangles distinguish unresolved exposure or safety questions. The combined exposure/formulation column includes studies without measured skin concentrations. Selected study-level checks are reported in Table S4; the matrix is not a substitute for them.

Material	Key references	Scope note
PUVA protocol	Elmets et al., 2019; Ling et al., 2016; Bethea et al., 1999; Olennikov, 2023; Parrish et al., 1974; Tegeder et al., 2002; Berneburg et al., 2013; Stern, 2012	Defined clinical photosensitizers; mainly methoxsalen. Clinical data do not validate a crude plant preparation.
Osthol	Matsuda et al., 2002; Hu et al., 2023; Yang et al., 2016; Kordulewska and Król-Grzymała, 2024; He et al., 2025; Naeem et al., 2023; Kim et al., 2016	Includes topical osthol during oxazolone induction, oral and local-injection studies, and culture assays. The topical carrier and dose basis are incompletely specified; no viable human-skin exposure-response.
Imperatorin	Zhao et al., 2022; Baek et al., 2000; Wang and Zhu, 2026; Tsai et al., 2021; Ljunggren, 1977	Psoriasis experiments used systemic dosing and an *A. hirsutiflora* isolate (>97% purity); rosacea used a commercial compound in a short topical LL-37 model. No matched topical UVA margin.
Xanthotoxol	Zhao et al., 2022; Gao et al., 2023	Non-topical dosing; mechanistic work includes spinal signaling rather than exclusively skin-local action.
Umbelliferone	Olennikov, 2023; Kim et al., 2024b; Kim et al., 2024a; Lim et al., 2019	Healthy-volunteer pigmentation measurements and oral diabetic-mouse wound closure; commercial identity is separate from plant provenance.
Decursin / decursinol angelate	Ahn et al., 2008; Han et al., 2018; Park et al., 2012; Li et al., 2013; Zhang et al., 2015; Kim et al., 2015; Chang et al., 2021; Chang et al., 2022	Parent compounds and decursinol must be distinguished; parent–metabolite PK is not direct skin-exposure evidence.

Material	Key references	Scope note
Esculetin	Olennikov, 2023; Chen et al., 2018; Khalil et al., 2024	Khalil: commercial esculetin >98%; formulation/release and short UV-lesion data. Skin exposure unmeasured; UV spectrum and application-dose basis unresolved. No Apiaceae batch link.
Scopoletin / isoscopoletin	Zhao et al., 2022; Seo et al., 2024; Bak et al., 2022	Mouse efficacy belongs to *Lycium extract. Isolated-compound findings are cellular.*
Peucedanum japonicum extract	Kim et al., 2025	Traceability refers to a named botanical extract, not complete active-constituent identification.
Umbelliprenin	Barthomeuf et al., 2008	Isolated from *Ferula; melanoma-cell findings only.*
Khellin–UVA	Ortel et al., 1988; Duschet et al., 1989; de Leeuw et al., 2003	Historical non-coumarin comparator; small human studies and hepatic safety signals.

References for supplementary tables

Ahn, M.J.; Lee, M.K.; Kim, Y.C.; Sung, S.H. The simultaneous determination of coumarins in *Angelica gigas root by high performance liquid chromatography–diode array detector coupled with electrospray ionization/mass spectrometry. J. Pharm. Biomed. Anal. 2008, 46, 258–266. https://doi.org/10.1016/j.jpba.2007.09.020*

Baek, N.I.; Ahn, E.M.; Kim, H.Y.; Park, Y.D. Furanocoumarins from the root of *Angelica dahurica. Arch. Pharm. Res. 2000, 23, 467–470. https://doi.org/10.1007/BF02976574*

Bak, S.G.; Lim, H.J.; Won, Y.S.; Lee, S.; Cheong, S.H.; Lee, S.J.; Bae, E.Y.; Lee, S.W.; Lee, S.J.; Rho, M.C. Regulatory effects of *Lycium barbarum extract and isolated scopoletin on atopic dermatitis-like skin inflammation. BioMed Res. Int. 2022, 2022, 2475699. https://doi.org/10.1155/2022/2475699*

Barthomeuf, C.; Lim, S.; Iranshahi, M.; Chollet, P. Umbelliprenin from *Ferula szowitsiana inhibits the growth of human M4Beu metastatic pigmented malignant melanoma cells through cell-cycle arrest in G1 and induction of caspase-dependent apoptosis. Phytomedicine 2008, 15, 103–111. https://doi.org/10.1016/j.phymed.2007.04.001*

Berneburg, M.; Herzinger, T.; Rampf, J.; Hoetzenecker, W.; Guenova, E.; Meisner, C.; Maetzke, J.; Schaefer, T.; Eberlein, B.; Scharffetter-Kochanek, K.; Rocken, M. Efficacy of bath psoralen plus ultraviolet A (PUVA) vs. system PUVA in psoriasis: a prospective, open, randomized, multicentre study. Br. J. Dermatol. 2013, 169, 704–708. https://doi.org/10.1111/bjd.12466

Bethea, D.; Fullmer, B.; Syed, S.; Seltzer, G.; Tiano, J.; Rischko, C.; Gillespie, L.; Brown, D.; Gasparro, F.P. Psoralen photobiology and photochemotherapy: 50 years of science and medicine. J. Dermatol. Sci. 1999, 19, 78–88. https://doi.org/10.1016/S0923-1811(98)00064-4

Chang, S.N.; Khan, I.; Kim, C.G.; Park, S.M.; Choi, D.K.; Lee, H.; Hwang, B.S.; Kang, S.C.; Park, J.G. Decursinol angelate arrest melanoma cell proliferation by initiating cell death and tumor shrinkage via induction of apoptosis. Int. J. Mol. Sci. 2021, 22, 4096. https://doi.org/10.3390/ijms22084096

Chang, S.N.; Khan, I.; Kim, C.G.; Park, S.M.; Choi, D.K.; Lee, H.; Hwang, B.S.; Kang, S.C.; Park, J.G. Correction: Chang et al. Decursinol angelate arrest melanoma cell proliferation by initiating cell death and tumor shrinkage via induction of apoptosis. Int. J. Mol. Sci. 2021, 22, 4096. Int. J. Mol. Sci. 2022, 23, 1629. https://doi.org/10.3390/ijms23031629

Chen, Y.; Zhang, Q.; Liu, H.; Lu, C.; Liang, C.L.; Qiu, F.; Han, L.; Dai, Z. Esculetin ameliorates psoriasis-like skin disease in mice by inducing CD4+Foxp3+ regulatory T cells. Front. Immunol. 2018, 9, 2092. https://doi.org/10.3389/fimmu.2018.02092

Chinese Pharmacopoeia Commission. Pharmacopoeia of the People's Republic of China, 2020 ed.; Volume I; China Medical Science and Technology Press: Beijing, China, 2020; monographs for Cnidii Fructus and Angelicae Dahuricae Radix; pp. 328–329 (Cnidii Fructus) and 109–110 (Angelicae Dahuricae Radix). (In Chinese)

de Leeuw, J.; van der Beek, N.; Maierhofer, G.; Neugebauer, W.D. A case study to evaluate the treatment of vitiligo with khellin encapsulated in L-phenylalanin stabilized phosphatidylcholine liposomes in combination with ultraviolet light therapy. Eur. J. Dermatol. 2003, 13, 474–477. https://pubmed.ncbi.nlm.nih.gov/14693493/

Duschet, P.; Schwarz, T.; Pusch, M.; Gschnait, F. Marked increase of liver transaminases after khellin and UVA therapy. J. Am. Acad. Dermatol. 1989, 21, 592–593. https://doi.org/10.1016/S0190-9622(89)80248-8

Elmets, C.A.; Lim, H.W.; Stoff, B.; Connor, C.; Cordoro, K.M.; Lebwohl, M.; Armstrong, A.W.; Davis, D.M.R.; Elewski, B.E.; Gelfand, J.M.; Gordon, K.B.; Gottlieb, A.B.; Kaplan, D.H.; Kavanaugh, A.; Kiselica, M.; Kivelevitch, D.; Korman, N.J.; Kroshinsky, D.; Leonardi, C.L.; Lichten, J.; Mehta, N.N.; Paller, A.S.; Parra, S.L.; Pathy, A.L.; Farley Prater, E.A.; Rupani, R.N.; Siegel, M.; Strober, B.E.; Wong, E.B.; Wu, J.J.; Hariharan, V.; Menter, A. Joint American Academy of Dermatology–National Psoriasis Foundation guidelines of care for the management and treatment of psoriasis with phototherapy. J. Am. Acad. Dermatol. 2019, 81, 775–804. https://doi.org/10.1016/j.jaad.2019.04.042

Gao, X.; Yang, Y.; Zhu, J.; Zhang, Y.; Wang, C.; Wang, Z.; Mi, W.; Du, L. Xanthotoxol relieves itch in mice via suppressing spinal GRP/GRPR signaling. Eur. J. Pharmacol. 2023, 960, 176147. https://doi.org/10.1016/j.ejphar.2023.176147

Han, J.; Jin, W.; Ho, N.A.; Hong, J.; Kim, Y.J.; Shin, Y.; Lee, H.; Suh, J.W. Decursin and decursinol angelate improve wound healing by upregulating transcription of genes encoding extracellular matrix remodeling proteins, inflammatory cytokines, and growth factors in human keratinocytes. Biochem. Biophys. Res. Commun. 2018, 499, 979–984. https://doi.org/10.1016/j.bbrc.2018.04.031

He, S.; Liang, X.; Chen, W.; Nima, Y.; Li, Y.; Gu, Z.; Lai, S.; Zhong, F.; Qiu, C.; Mo, Y.; Tang, J.; Wu, G. Osthole ameliorates chronic pruritus in 2,4-dichloronitrobenzene-induced atopic dermatitis by inhibiting IL-31 production. Chin. Herb. Med. 2025, 17, 368–379. https://doi.org/10.1016/j.chmed.2024.01.003

Hu, X.; Zhou, Y.; Shi, J.; Qi, M.; Li, X.; Yang, Y.; Zhu, C.; Wang, C.; Tang, Z.; Ma, Y.; Yu, G. Osthole relieves skin damage and inhibits chronic itch through modulation of Akt/ZO-3 pathway in atopic dermatitis. Eur. J. Pharmacol. 2023, 947, 175649. https://doi.org/10.1016/j.ejphar.2023.175649

Khalil, R.M.; Abdelhameed, M.F.; Abou Taleb, S.; El-Saied, M.A.; Shalaby, E.S. Preparation and characterisation of esculetin-loaded nanostructured lipid carriers gels for topical treatment of UV-induced psoriasis. Pharm. Dev. Technol. 2024, 29, 886–898. https://doi.org/10.1080/10837450.2024.2407854

Kim, B.S.; Seo, H.; Kim, H.J.; Bae, S.M.; Son, H.N.; Lee, Y.J.; Ryu, S.; Park, R.W.; Nam, J.O. Decursin from *Angelica gigas Nakai inhibits B16F10 melanoma growth through induction of apoptosis. J. Med. Food 2015, 18, 1121–1127. https://doi.org/10.1089/jmf.2014.3397*

Kim, D.J.; Jung, M.S.; Jin, H.U.; Kim, M.S.; An, C.E. Anti-melanogenic effects of umbelliferone: in vitro and clinical studies. Molecules 2024, 29, 5571. https://doi.org/10.3390/molecules29235571

Kim, D.Y.; Kang, Y.H.; Kang, M.K. Umbelliferone alleviates impaired wound healing and skin barrier dysfunction in high glucose-exposed dermal fibroblasts and diabetic skins. J. Mol. Med. 2024, 102, 1457–1470. https://doi.org/10.1007/s00109-024-02491-z

Kim, J.H.; Han, K.S.; Kang, E.S.; Park, J.H.; Cho, B.O.; Choi, J.H. *Peucedanum japonicum Thunberg root extract inhibits atopic dermatitis-like skin symptoms. Allergol. Immunopathol. (Madr) 2025, 53, 12–29. https://doi.org/10.15586/aei.v53i5.1399*

Kim, S.B.; Kim, C.T.; Liu, Q.; Jo, Y.H.; Choi, H.J.; Hwang, B.Y.; Kim, S.K.; Lee, M.K. Optimization of extraction conditions for osthol, a melanogenesis inhibitor from *Cnidium monnieri fruits. Pharm. Biol. 2016, 54, 1373–1379. https://doi.org/10.3109/13880209.2015.1078382*

Kordulewska, N.K.; Król-Grzymała, A. The effect of osthole on transient receptor potential channels: a possible alternative therapy for atopic dermatitis. J. Inflamm. Res. 2024, 17, 881–898. https://doi.org/10.2147/JIR.S425978

Li, L.; Zhang, J.; Xing, C.; Kim, S.H.; Lü, J. Single oral dose pharmacokinetics of decursin, decursinol angelate, and decursinol in rats. Planta Med. 2013, 79, 275–280. https://doi.org/10.1055/s-0032-1328202

Lim, J.Y.; Lee, J.H.; Lee, D.H.; Lee, J.H.; Kim, D.K. Umbelliferone reduces the expression of inflammatory chemokines in HaCaT cells and DNCB/DFE-induced atopic dermatitis symptoms in mice. Int. Immunopharmacol. 2019, 75, 105830. https://doi.org/10.1016/j.intimp.2019.105830

Ling, T.C.; Clayton, T.H.; Crawley, J.; Exton, L.S.; Goulden, V.; Ibbotson, S.; McKenna, K.; Mohd Mustapa, M.F.; Rhodes, L.E.; Sarkany, R.; Dawe, R.S. British Association of Dermatologists and British Photodermatology Group guidelines for the safe and effective use of psoralen–ultraviolet A therapy 2015. Br. J. Dermatol. 2016, 174, 24–55. https://doi.org/10.1111/bjd.14317

Ljunggren, B. Psoralen photoallergy caused by plant contact. Contact Dermatitis 1977, 3, 85–90. https://doi.org/10.1111/j.1600-0536.1977.tb03604.x

Matsuda, H.; Tomohiro, N.; Ido, Y.; Kubo, M. Anti-allergic effects of Cnidii Monnieri Fructus (dried fruits of *Cnidium monnieri) and its major component, osthol. Biol. Pharm. Bull. 2002, 25, 809–812. https://doi.org/10.1248/bpb.25.809*

Naeem, M.; Iqbal, T.; Yousuf, M.; Nawaz, Z.; Hussain, S.; Alamri, A.S.; Galanakis, C.M.; Ali, A. Preparation, optimization and evaluation of osthole transdermal therapeutic system. An. Acad. Bras. Cienc. 2023, 95, e20221023. https://doi.org/10.1590/0001-3765202320221023

Olennikov, D.N. Coumarins of Lovage Roots (*Levisticum officinale* W.D.J.Koch): LC-MS Profile, Quantification, and Stability during Postharvest Storage. Metabolites 2023, 13, 3. https://doi.org/10.3390/metabo13010003

Ortel, B.; Tanew, A.; Hönigsmann, H. Treatment of vitiligo with khellin and ultraviolet A. J. Am. Acad. Dermatol. 1988, 18, 693–701. https://doi.org/10.1016/S0190-9622(88)70092-4

Park, H.; Kim, B.; Oh, J.H.; Kim, Y.; Lee, Y.J. First-pass metabolism of decursin, a bioactive compound of *Angelica gigas, in rats. Planta Med. 2012, 78, 909–913. https://doi.org/10.1055/s-0031-1298517*

Parrish, J.A.; Fitzpatrick, T.B.; Tanenbaum, L.; Pathak, M.A. Photochemotherapy of psoriasis with oral methoxsalen and longwave ultraviolet light. N. Engl. J. Med. 1974, 291, 1207–1211. https://doi.org/10.1056/NEJM197412052912301

Pathak, M.A.; Fitzpatrick, T.B. The evolution of photochemotherapy with psoralens and UVA (PUVA): 2000 BC to 1992 AD. J. Photochem. Photobiol. B 1992, 14, 3–22. https://doi.org/10.1016/1011-1344(92)85080-E

Seo, D.Y.; Park, J.W.; Kim, S.H.; Oh, S.R.; Han, S.B.; Kwon, O.K.; Ahn, K.S. Effect of isoscopoletin on cytokine expression in HaCaT keratinocytes and RBL-2H3 basophils: preliminary study. Int. J. Mol. Sci. 2024, 25, 6908. https://doi.org/10.3390/ijms25136908

Stern, R.S. The risk of squamous cell and basal cell cancer associated with psoralen and ultraviolet A therapy: a 30-year prospective study. J. Am. Acad. Dermatol. 2012, 66, 553–562. https://doi.org/10.1016/j.jaad.2011.04.004

Tegeder, I.; Bräutigam, L.; Podda, M.; Meier, S.; Kaufmann, R.; Geisslinger, G.; Grundmann-Kollmann, M. Time course of 8-methoxypsoralen concentrations in skin and plasma after topical (bath and cream) and oral administration of 8-methoxypsoralen. Clin. Pharmacol. Ther. 2002, 71, 153–161. https://doi.org/10.1067/mcp.2002.121908

Tsai, Y.F.; Chen, C.Y.; Lin, I.W.; Leu, Y.L.; Yang, S.C.; Syu, Y.T.; Chen, P.J.; Hwang, T.L. Imperatorin alleviates psoriasiform dermatitis by blocking neutrophil respiratory burst, adhesion, and chemotaxis through selective phosphodiesterase 4 inhibition. Antioxid. Redox Signal. 2021, 35, 885–903. https://doi.org/10.1089/ars.2019.7835

Wang, X.; Zhu, Z. Imperatorin mitigates rosacea-like inflammation by modulating the crosstalk between JNK1 and STAT1. FASEB J. 2026, 40, e71641. https://doi.org/10.1096/fj.202503868RR

Yang, N.N.; Shi, H.; Yu, G.; Wang, C.M.; Zhu, C.; Yang, Y.; Yuan, X.L.; Tang, M.; Wang, Z.L.; Gegen, T.; He, Q.; Tang, K.; Lan, L.; Wu, G.Y.; Tang, Z.X. Osthole inhibits histamine-dependent itch via modulating TRPV1 activity. Sci. Rep. 2016, 6, 25657. https://doi.org/10.1038/srep25657

Zhang, J.; Li, L.; Hale, T.W.; Chee, W.; Xing, C.; Jiang, C.; Lü, J. Single oral dose pharmacokinetics of decursin and decursinol angelate in healthy adult men and women. PLoS One 2015, 10, e0114992. https://doi.org/10.1371/journal.pone.0114992

Zhao, H.; Feng, Y.L.; Wang, M.; Wang, J.J.; Liu, T.; Yu, J. The *Angelica dahurica: a review of traditional uses, phytochemistry and pharmacology. Front. Pharmacol. 2022, 13, 896637. https://doi.org/10.3389/fphar.2022.896637*

Table S4 Study-level evidence underlying candidate interpretation

The eleven selected primary reports were inspected in full. A denotes direct support for the stated details in the available primary text; it is a source-access label, not a formal risk-of-bias rating. Supplementary files for Hu et al., Tsai et al., and Khalil et al. were not available for direct inspection. Dose and sample-size units belong to each experiment and must not be pooled across models. Full references appear above.

**Study and actual material**	**Model and route**	**Design and observed outcome**	**Exposure and limits of interpretation**
Matsuda et al., 2002 [A] Authenticated *Cnidium fruit; voucher CM-99001; 70% ethanol extract and spectroscopically characterized osthol from the same material.*	Contact-hypersensitivity mouse experiments. Oral extract 200/500 mg/kg; osthol 20 mg/kg in DNFB and 100 mg/kg in picryl-chloride experiments. Timing differed by model.	Oral diphenhydramine/prednisolone reference arms; endpoint-specific n approximately 8–14/group. Extract and osthol reduced ear swelling; scratching was tested with the extract.	No measured viable-skin concentration or matched UVA test in these experiments. Supports one extract–constituent link, not all traditional preparations or topical clinical efficacy.
Yang et al., 2016 [A] Commercial osthol.	Acute pruritogen challenge and mouse dorsal-root-ganglion neurons. Local subcutaneous injection, 50 microlitres/site, 30 min before challenge; methods list 0.01, 1, and 30 micromolar.	Vehicle and pruritogen controls; blinded behavioral scoring; histamine n=6/group, other assays differ. AMG9810 is a neuronal mechanistic comparator. Histamine-related scratching and calcium responses fell; chloroquine scratching did not.	Injection-site concentration is not a measured skin-tissue concentration; no topical barrier passage tested. Acute pretreatment. Some investigators also contributed to Hu et al.; complementary reports do not establish independent replication of the same regimen.
Hu et al., 2023 [A] Commercial osthol, Yuan Ye S26004; purity and batch not reported in the main text.	Male C57BL/6J oxazolone dermatitis; HaCaT experiments. Three AD patient biopsies were observational. Topical 15/45 mg/kg, twice daily for seven days from first oxazolone challenge. Per-application versus daily dose basis and carrier not specified.	Vehicle and two osthol groups, n=6/group; no standard active drug. Separate LY294002 arm, n=8/group. Dermatitis allocation/blinding not reported in the main text. Lower lesion scores/scratching and altered ZO-3/p-Akt signals. ZO-3 knockdown reduced HaCaT resistance in a separate experiment.	No measured skin drug concentration, osthol-associated resistance/TEWL rescue, or matched UVA test. 20/40 micromolar reduced HaCaT viability at 24 h; 10 micromolar was used for ZO-3 experiments. Induction-overlap activity, not established-lesion therapy. No osthol-plus-ZO-3 knockdown necessity test. Table S1 was not included in the downloaded main text.
Kordulewska and Król-Grzymała, 2024 [A] Sigma-Aldrich osthol Y0001207; ethanol stock; clobetasol stock in DMSO.	NHEK and organotypic skin stimulated with histamine or LPS. Apical osthol 0.0625–0.5 mg/mL after 3-h stimulation; 72-h incubation. Selected result panels use 0.125/0.5 mg/mL.	Clobetasol positive control; assay replicates reported, without a clear donor-level independent n in the inspected methods. Cytokine/receptor-expression changes; no behavioral itch endpoint.	Nominal culture dose, not measured viable-skin drug concentration; no matched UVA assay. Active control supports an experimental comparison; different solvents, nominal dose and replicate structure limit topical potency inference.
He et al., 2025 [A] Aladdin osthol, stated 99% purity.	Male C57BL/6J DNCB dermatitis and stimulated HaCaT cells. 5/25/50 mg/kg; gavage stated in Discussion. Full osthol frequency and schedule incompletely specified in Methods.	DNCB/vehicle arms and topical tacrolimus 0.03%; n=6 for mouse figures. Three scorers described; randomization/blinding not specified in inspected text. Dermatitis, scratching and epidermal changes; cellular PPAR antagonist experiments.	No quantified skin osthol or UVA margin. Oral osthol versus topical tacrolimus is not a topical product comparison; causal PPAR tests were cellular.
Naeem et al., 2023 [A] Commercial osthol; microemulsion made with 2% loading, then incorporated into Carbomer gel.	Excised rabbit-skin permeation; rabbit irritation and formalin edema. Local gel application; edema regimen once daily for seven days. Final applied mass and gel loading require clearer reporting.	Conventional gel also contained osthol; untreated reference. Six rabbits/group for edema; separate irritation observations. Improved permeation and edema readouts; short local-tolerance evidence.	Receiver permeation measured; viable-skin retention and plasma PK not established. No matched UVA test. Delivery experiment, not an AD efficacy model; formulation effects and systemic bioavailability are different claims.

Study and actual material	Model and route	Design and observed outcome	Exposure and limits of interpretation
Tsai et al., 2021 [A] Imperatorin isolated by the authors from *Angelica hirsutiflora*; stated purity >97%.	Healthy-donor neutrophils; imiquimod in BALB/c and C57BL/6 mice; IL-23 in C57BL/6 mice.<br>Imiquimod: tail-vein 1/5/15 mg/kg in BALB/c and 15 mg/kg in C57BL/6, daily days 3–6. IL-23: intraperitoneal 15 mg/kg on cytokine-injection days, every two days over 12 days.	Six mice/group; sham and vehicle controls, 40 microlitres 50% DMSO/50% Tween 80. No active antipsoriatic drug arm. Dermatitis randomization/blinding not reported in the main text.<br>Reduced psoriasiform and neutrophil endpoints; purified PDE4 subtype assays and cellular PKA-inhibitor reversal support pathway involvement.	Systemic routes; no topical formulation, measured skin PK, or paired UVA test. Main-text Figures 8–11 were inspected; supplementary files were not included in the download.<br>Early intervention/co-induction, not established chronic disease. No dermatitis target-dependence experiment; off-target effects remain possible. Two models are not independent replication.
Wang and Zhu, 2026 [A] Aladdin imperatorin I114064; purity >98%.	Acute LL-37 mouse lesions; HaCaT and HEK293T mechanistic experiments.<br>20 mg/kg intraperitoneally before induction; topical 3% w/w in vaseline (30 mg/g formulation) twice daily for three days.	n=6/group; vehicle controls; no active rosacea drug. Allocation/blinding not specified in inspected methods.<br>Redness, lesion area and histology; siRNA, Co-IP and thermal-shift observations.	No measured viable-skin level or matched UVA margin. Residual ethanol in ointment not specified.<br>Short induction regimen, not established chronic disease. Target participation is better supported than specific direct dual binding.
Kim et al., 2024a [A] Commercial umbelliferone; 0.0025% ampoule (25 micrograms/mL as reported) in light-resistant containers.	12 healthy Korean adults, 20–59 years; complementary cell assays.<br>Randomized split-face application twice daily for four weeks.	Identical containers; double-blind vehicle comparison; no clinical active drug. Cell assays included arbutin.<br>Small pigmentation-index changes; disease efficacy and durability unestablished.	No measured viable-skin PK. Sunlight stability loss was measured; not a photosafety assay.<br>Small healthy cohort; no diagnosis-defined trial. Authors employed by a cosmetics company. Figure captions and clinical statistical-unit wording need clarification.
Kim et al., 2024b [A] Commercial umbelliferone from Sigma-Aldrich.	STZ/high-fat-diet diabetic mice and high-glucose human dermal fibroblasts.<br>10 mg/kg orally over a reported 10-week course; omitted on wounding day and next two days. Four 6-mm wounds/mouse; follow-up to day 18.	n=9–10/subgroup; diabetic allocation described as random; water-treated diabetic and non-diabetic controls.<br>Faster wound closure and matrix/barrier-associated changes.	No measured tissue drug concentration or matched UVA test.<br>Experimental excisions; Methods specify 10 weeks whereas the Figure 6 legend states four weeks. Multiple wounds per animal require animal-level analysis; no human standard-care adjunct comparison.
Khalil et al., 2024 [A] Commercial esculetin >98% (Chengdu Herbpurify); 0.1% w/v in Table1. F2 uses Emulgin B2; F5 uses Tween 80; gum Arabic gel.	Male Wistar rat UV-irradiation lesions. UV-B is stated alongside 254 nm; actual spectrum is unresolved.<br>Topical DS/F2/F5 labelled 1%/2%, once daily for four days, reported 12 h after irradiation. Applied amount and relation to 0.1% loading unclear.	Eight randomized groups, n=10; histology n=3/group, five fields/rat. Normal/model and free-drug controls; no empty carrier or active drug. Blinding not stated.<br>F2 lowered TNF-alpha/CD34 and improved selected histological outcomes; F5 did not significantly lower TNF-alpha.	Dialysis-membrane release, not skin permeation. No measured skin retention/PK or paired UVA test.<br>Short UV model, uncertain spectrum/dose basis and unresolved vehicle contribution. LSD/Tukey and acute-toxicity reporting differ; supplements not inspected.

A reusable study extraction, source locators, and reference numbers corresponding to the main manuscript are supplied in Table_S4_study_evidence.csv. Missing details in an inspected main text are distinguished from information in unavailable attachments or other uninspected references. These selected checks do not constitute full-text reassessment of every reference in the review.

Table S5 Record of literature searches and evidence acquisition

The literature collection for this narrative review was assembled in stages, supplemented by targeted searches and by full-text checking of selected studies (main text, Section 2.1). Part A lists the steps that are documented in project records, with the limits of each record. Part B sets out the search concepts as a search strategy compiled afterwards; its historical execution could not be confirmed, and it was not re-run for this revision. Part C lists the scope criteria currently applied

to full texts; it is not a tally of historical exclusions. The total number of records retrieved, the number after removal of duplicates, and the numbers excluded at title/abstract and full-text stages were not retained and are therefore not reported. Literature screening was performed by two authors (J.G. and Y.Z.), assisted by the generative AI tool described in Section 2.5 of the main text.

A. Documented searches and evidence-acquisition steps

Date and basis	Source or tool	Recovered search concepts or query	Purpose	Verifiable result	Limitations of the record
27 July 2026; project work report	PubMed, title-level search	Coumarin or furocoumarin combined with skin, dermatology, psoriasis, vitiligo, or dermatitis; reviews	Check for existing reviews on the same topic	4 records returned	Full Boolean string, field tags, and filters not recovered; original search response not retained. Not a total for the review.
5 September 2026; project log and cached search results	Web searches; OpenAlex-assisted discovery	Not fully recovered	Targeted update: identify the closest reviews and check selected primary studies	Saved OpenAlex response: 20 returned items	Number of returned items in one saved response; not the total number of database hits or of included studies. Not an exhaustive repeat search.
5 September 2026; project log	Comparison with reviews on the same topic	Not applicable	Compare the scope and contribution of this review	10 reviews compared: key sections of 7 read in full text, 3 read in part	Review comparison set; separate from the primary studies in Table S4 and not counted with them.
Successive project versions and logs	Full-text acquisition and checking	Not applicable	Detailed study-level checks recorded in Table S4	Initially 10 reports: 8 assessed from main texts; Hu et al. and Tsai et al. at abstract level. Full texts of Hu et al. and Tsai et al. then obtained (10 reports). Full text of Khalil et al. then obtained and assessed (11 reports).	The set was formed stepwise; it was not set in advance and was not selected from a complete pool of candidate studies by a predefined rule.
Project log (one full-text recovery batch)	Full-text files	Not applicable	Obtain full texts for detailed checking	4 PDF files corresponding to 3 independent papers, including one duplicate file of Hu et al.	Applies to this batch only; not a deduplication count for the review.

B. Search strategy compiled afterwards to set out the search concepts (historical execution not confirmed; not re-run)

Block	Concept	Search string (PubMed syntax)
1	Plant-led	(Apiaceae[MeSH Terms] OR Apiaceae OR Umbelliferae OR Angelica OR Cnidium OR Peucedanum OR Heracleum OR Pastinaca OR Ammi OR Ferula OR Levisticum) AND (coumarin* OR furanocoumarin* OR furocoumarin* OR psoralen*) AND (skin OR dermat* OR psoria* OR eczema OR prurit* OR itch* OR rosacea OR vitiligo OR pigment* OR melanoma OR wound* OR "mycosis fungoides" OR topical OR transdermal OR percutaneous OR phototox* OR photosensiti*)

Block	Concept	Search string (PubMed syntax)
2	Compound-led, without plant restriction	(methoxsalen OR xanthotoxin OR 8-methoxypsoralen OR bergapten OR 5-methoxypsoralen OR imperatorin OR xanthotoxol OR osthol OR osthole OR umbelliferone OR scopoletin OR isoscopoletin OR esculetin OR decursin OR "decursinol angelate" OR umbelliprenin) AND (skin OR dermat* OR psoria* OR eczema OR prurit* OR itch* OR rosacea OR vitiligo OR pigment* OR melanoma OR wound* OR "mycosis fungoides" OR topical OR transdermal OR percutaneous OR phototox* OR photosensiti*)
3	Photochemotherapy and photosafety	(PUVA OR "psoralen plus ultraviolet A" OR photochemotherapy OR phytophotodermatitis OR photoallerg* OR phototox*) AND (psoralen* OR furanocoumarin* OR furocoumarin* OR methoxsalen OR bergapten)

C. Scope criteria applied to full texts (current criteria; not a historical tally of exclusions)

Studies were not used as evidence when they were:
General antioxidant or enzyme screens without a skin-relevant model
Docking-only or other purely in silico studies
Experiments without a dermatological endpoint or connection
Reports in which the test material could not be identified
Duplicate reports of the same dataset (the most complete report was used)
